# Urine-mediated suppression of *Klebsiella pneumoniae* mucoidy is counteracted by spontaneous Wzc variants altering capsule chain length

**DOI:** 10.1128/msphere.00288-23

**Published:** 2023-08-23

**Authors:** Saroj Khadka, Brooke E. Ring, Ryan S. Walker, Lindsey R. Krzeminski, Drew A. Pariseau, Matthew Hathaway, Harry L. T. Mobley, Laura A. Mike

**Affiliations:** 1 Medical Microbiology and Immunology, University of Toledo, Toledo, Ohio, USA; 2 Microbiology and Immunology, University of Michigan, Ann Arbor, Michigan, USA; University of Kentucky College of Medicine, Lexington, Kentucky, USA

**Keywords:** *Klebsiella pneumoniae*, mucoidy, hypermucoviscosity, bacterial pathogenesis, hypervirulence, urinary tract infections, capsule biosynthesis, capsule chain length, Wzc, tyrosine kinase, phosphotyrosine, phosphosignaling

## Abstract

*Klebsiella pneumoniae* is a hospital-associated pathogen primarily causing urinary tract infections (UTIs), pneumonia, and septicemia. Two challenging lineages include the hypervirulent strains, causing invasive community-acquired infections, and the carbapenem-resistant classical strains, most frequently isolated from UTIs. While hypervirulent strains are often characterized by a hypermucoid phenotype, classical strains usually present with low mucoidy. Since clinical UTI isolates tend to exhibit limited mucoidy, we hypothesized that environmental conditions may drive *K. pneumoniae* adaptation to the urinary tract and select against mucoid isolates. We found that both hypervirulent *K. pneumoniae* and classical *Klebsiella* UTI isolates significantly suppressed mucoidy when cultured in urine without reducing capsule abundance. A genetic screen identified secondary mutations in the *wzc* tyrosine kinase that overcome urine-suppressed mucoidy. Over-expressing Wzc variants *in trans* was sufficient to boost mucoidy in both hypervirulent and classical *Klebsiella* UTI isolates. Wzc is a bacterial tyrosine kinase that regulates capsule polymerization and extrusion. Although some Wzc variants reduced Wzc phospho-status, urine did not alter Wzc phospho-status. Urine does, however, increase *K. pneumoniae* capsule chain length diversity and enhance cell-surface attachment. The identified Wzc variants counteract urine-mediated effects on capsule chain length and cell attachment. Combined, these data indicate that capsule chain length correlates with *K. pneumoniae* mucoidy and that this extracellular feature can be fine-tuned by spontaneous Wzc mutations, which alter host interactions. Spontaneous Wzc mutation represents a global mechanism that could fine-tune *K. pneumoniae* niche-specific fitness in both classical and hypervirulent isolates.

**IMPORTANCE:**

*Klebsiella pneumoniae* is high-priority pathogen causing both hospital-associated infections, such as urinary tract infections, and community-acquired infections. Clinical isolates from community-acquired infection are often characterized by a tacky, hypermucoid phenotype, while urinary tract isolates are usually not mucoid. Historically, mucoidy was attributed to capsule overproduction; however, recent reports have demonstrated that *K. pneumoniae* capsule abundance and mucoidy are not always correlated. Here, we report that human urine suppresses *K. pneumoniae* mucoidy, diversifies capsule polysaccharide chain length, and increases cell surface association. Moreover, specific mutations in the capsule biosynthesis gene, *wzc*, are sufficient to overcome urine-mediated suppression of mucoidy. These Wzc variants cause constitutive production of more uniform capsular polysaccharide chains and increased release of capsule from the cell surface, even in urine. These data demonstrate that *K. pneumoniae* regulates capsule chain length and cell surface attachment in response host cues, which can alter bacteria-host interactions.

## INTRODUCTION

*Klebsiella pneumoniae* is a Gram-negative pathogen responsible for approximately 10% of all nosocomial infections and is the fourth deadliest bacterial species in the world (1, 2). Within the hospital setting, *Klebsiella* spp. cause 23% of all urinary tract infections (UTIs), 14% of all surgical-site infections, 12% of all pneumonia cases, and 8% of all bloodstream infections (1). In the community setting, *K. pneumoniae* is the second most common cause of uncomplicated UTI after *Escherichia coli* (3). *K. pneumoniae* isolates that pose a major threat to human health include carbapenem-resistant classical strains (CR-cKp) and hypervirulent strains (hvKp), which cause severe morbidities and high mortality (4 – 6). CR-cKp was first observed in 1996, and since then, it has been the major driving force disseminating the carbapenem resistance cassette throughout the *Enterobacteriaceae,* complicating the treatment of Gram-negative infections (7, 8). Of all healthcare-associated infections, CR-cKp is most commonly isolated from UTI cases, challenging the therapeutic choices of healthcare providers (9 – 11). In parallel, the incidence of hvKp is rising in the community setting and beginning to also appear in hospitals, where it was previously absent (12 – 15). HvKp is associated with invasive infections, especially pyogenic liver abscesses that disseminate to the eyes and brain, a pathogenesis uncommon for Gram-negative enteric bacteria (4, 5). Features associated with hvKp include hypermucoidy, *rmpA* overexpression, and K1 or K2 capsular polysaccharide (5, 12, 14, 15). Hypermucoid strains are very tacky and sediment poorly due to culture viscosity when centrifuged. Although these features are associated with hvKp, they are not sole determinants. For example, some hvKp do not encode K1 or K2 capsular polysaccharide (CPS), and some K1- or K2-encapsulated strains are not hypervirulent.

The historical model of *K. pneumoniae* hypervirulence has been that overproduction of CPS boosts mucoidy and promotes resistance to host defenses. This is due to observations that elevated *rmpA* expression increases CPS production and mucoidy and that the loss of CPS production eliminates mucoidy and virulence (5). However, recent studies have demonstrated that other factors also contribute to mucoidy. Dissection of the *rmp* locus identified its polycistronic structure consisting of the autoregulator *rmpA* and two downstream genes, *rmpC* and *rmpD*, that distinctly impact CPS and mucoidy (16). An *rmpC* deletion strain retains mucoidy despite reduced CPS (17). Correspondingly, an *rmpD* deletion strain loses mucoidy and retains full CPS production (18). Overexpressing *rmpD* in acapsular strains does not restore mucoidy, supporting the model that mucoidy requires CPS biosynthetic components (18). Our previous studies provide further support for this model, as we identified many genes that regulate CPS and/or mucoidy; however, we identified no genes that support mucoidy in acapsular strains (19). Recent work has demonstrated that RmpD does not affect CPS composition but rather interacts with the capsule biosynthesis protein, Wzc, and controls CPS chain length (20).

These previous studies were performed in the ATCC 43816 derivative, KPPR1. This strain is a model hypervirulent and mucoid strain that produces a K2 CPS, comprising glucose (Glc), mannose [Man (70% OAc)], and glucuronic acid (GlcA) (20). *K. pneumoniae* employs the Wzy-dependent pathway to synthesize their Group 1 CPS (21). Undecaprenyl diphosphate (UDP)-linked CPS subunits are assembled on the cytoplasmic side of the inner membrane by glycosyltransferases and then flipped to the periplasm by Wzx. There, Wzy polymerizes the growing capsular polysaccharide chain. Continued polymerization requires coordinated Wzc tyrosine autokinase and Wzb phosphatase activity (22). Wzc is an integral inner membrane protein that forms a trans-periplasmic complex with the outer membrane CPS export pore protein, Wza. Non-phosphorylated Wzc associates as octamers and then dissociates upon autophosphorylation (23). These protein dynamics coordinate CPS polymerization and Wza-mediated extrusion of the growing polysaccharide chain (23). Wzi then anchors the CPS to the cell surface by an unknown mechanism (24, 25). Previous work identified CR-cKp isolates with increased mucoidy and CPS production in clinical bloodstream isolates, along with non-mucoid and acapsular strains in clinical UTI isolates (26). None of the isolates encoded the *rmp* locus, distinct from KPPR1. Whole-genome sequencing (WGS) revealed that point mutations in *wzc* increased CPS production and mucoidy, which promoted phagocytosis resistance, enhanced dissemination, and increased mortality in animal models. Conversely, disruptions in *wbaP* eliminated CPS production and mucoidy, which improves epithelial cell invasion, biofilm formation, and persistence in the urinary tract (26). These data demonstrate that mutation of Wzc is an Rmp*-*independent pathway that can enhance mucoidy and invasive disease, while loss of CPS biosynthesis and mucoidy can improve persistence during UTI. Combined, these data suggest that mucoidy may provide niche-specific advantages or disadvantages.

We hypothesized that environmental signals may control *Klebsiella* adaptation to the urinary tract. We found that hypervirulent, *rmp*-encoding and non-mucoid, *rmp-*negative strains have significantly reduced mucoidy when cultured in urine and that growth in urine down-regulated *rmpA, rmpC,* and *rmpD* transcripts. We identified secondary mutations in the Wzc tyrosine kinase sufficient to overcome urine-mediated suppression of mucoidy. All Wzc variants are sufficient to increase mucoidy independent of RmpD in an *rmp*-encoding hypervirulent strain. Furthermore, some Wzc variants are sufficient to increase mucoidy in *rmp*-negative classical strains. Given that Wzc is known to have autokinase activity and regulate CPS chain length and secretion, we examined the effects of Wzc variants and urine on Wzc phospho-status and CPS chain length and localization. Although some Wzc variants affected Wzc phospho-status, all Wzc variants standardized CPS chain length, which overcame urine-induced diversification of CPS chain length. Altogether, these data indicate that human urine down-regulates *rmpD,* increasing cell-associated CPS chain length diversity in *K. pneumoniae,* and that spontaneous Wzc variants act down-stream of RmpD to restore mucoidy and regulate CPS chain length in urine.

## RESULTS

### Urine suppresses mucoidy without reducing CPS abundance

To examine the effect of the urinary tract environment on *K. pneumoniae* mucoidy and CPS production, five strains were selected: two hypervirulent laboratory strains, KPPR1 (rifampin-resistant ATCC 43816 derivative) and NTUH-K2044, and three classical strains recently isolated from clinical UTI cases, 616, 714, and 1346 (27 – 29). All three clinical UTI isolates were originally identified as *K. pneumoniae* by MALDI-TOF, but our Pathogenwatch analysis determined that although 714 is *K. pneumoniae*, 616 is *Klebsiella variicola,* and 1346 is *Klebsiella quasipneumoniae* (30 – 32). Nonetheless, all strains belong to the *K. pneumoniae* species complex and, combined, provide representative clinical isolates for each pathogenic species within the complex, along with classical and hypervirulent *K. pneumoniae sensu stricto* (33). Pathogenwatch analysis determined that 616 encodes KL114 with an unknown K type, 714 is predicted to produce K23 CPS, and 1346 is predicted to produce K1 CPS (30, 31, 34). Pathogenwatch analyses also reported that none of these clinical UTI isolates carry the *rmpADC* genes, known to regulate CPS biosynthesis and mucoidy in hypervirulent *K. pneumoniae* (17, 18).

**Fig 1:**
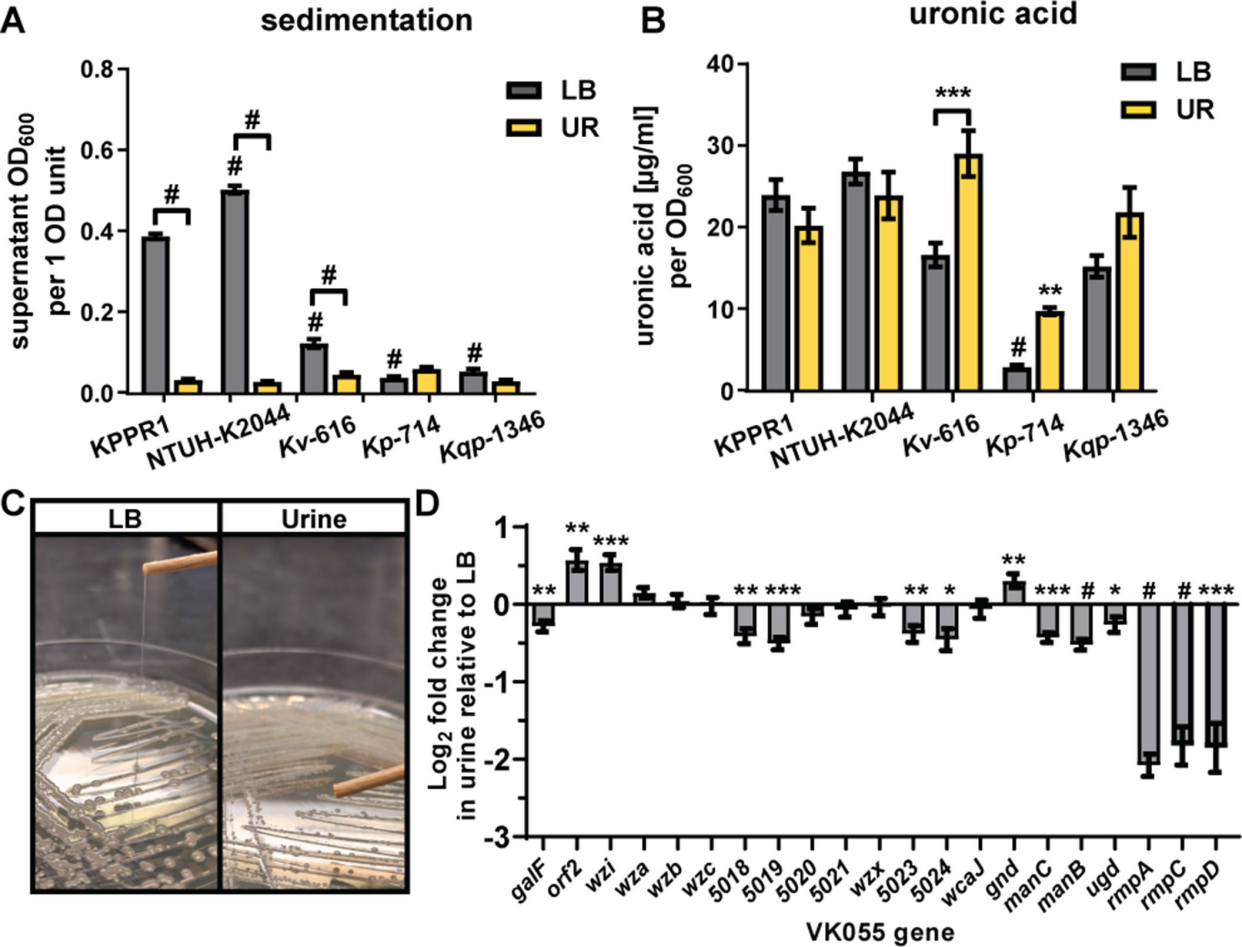
Urine suppresses mucoidy but not CPS biosynthesis. *K. pneumoniae* (*Kp*) strains KPPR1, NTUH-K2044, 714; *K. variicola* (*Kv*) 616; and *K. quasipneumoniae* (*Kqp*) 1364 were cultured in LB medium or sterile-filtered human urine (UR). (**A**) Mucoidy was determined by quantifying the supernatant OD_600_ after sedimenting 1 OD_600_ unit of culture at 1,000 × *g* for 5 min. (**B**) The uronic acid content of crude CPS extracts was determined after washing 1 OD_600_ unit of bacterial cells in PBS. (**C**) KPPR1 was cultured on LB agar or urine agar plates at 30°C overnight and then a string test was performed. (**D**) In addition, the relative abundance of each gene in the CPS biosynthesis and Rmp operons in UR relative to LB medium was determined by qRT-PCR and normalized to *gap2* transcript abundance. Data presented are the mean, and error bars represent the standard error of the mean. All experiments were performed ≥3 independent times, in triplicate. Statistical significance in panels **A** and **B** was determined using two-way ANOVA with a Bonferroni post-test to compare specific groups. Horizontal bars indicate LB medium versus UR comparisons, while symbols alone indicate comparisons to KPPR1 cultured in the same growth medium. In panel **D**, a Student’s *t* test was used to determine if each value was significantly different from 1.0. *P < 0.0332; **P < 0.0021; ***P < 0.0002; ^#^ *P* < 0.0001.

A standard quantitative measure of the mucoid (tacky colony) phenotype is the sedimentation assay. Mucoid bacteria produce a more viscous culture medium, which increases sedimentation resistance, i.e*.,* they do not sediment efficiently. To examine the effect of the urinary tract environment on *Klebsiella* mucoidy, the five selected isolates were cultured in rich medium (lysogeny broth [LB]) or pooled, sterile-filtered urine. As expected, the UTI isolates had less sedimentation resistance than the hypervirulent strain KPPR1 in LB medium (Fig. 1A). Unexpectedly, three of the strains had significantly less sedimentation resistance in urine than in LB medium, suggesting that either LB medium activates or urine suppresses the mucoid phenotype (Fig. 1A and C). We hypothesized that urine decreased cell-associated CPS, which could reduce sedimentation resistance. Bacterial cells were washed with PBS, and the uronic acid content of cell-associated CPS was quantified. Remarkably, culturing the strains in urine did not significantly reduce CPS production as measured by uronic acid content; in fact, it appeared to stimulate CPS production in UTI strain 616 (Fig. 1B). Notably, all strains had a similar CFU/mL count per OD_600_ value (Fig. S1). This indicates that the observed differences are not because of varied CPS abundance on OD_600_ readings. Combined, these results demonstrated that environmental conditions may regulate the mucoid phenotype independently of CPS and that *Klebsiella* mucoidy may be actively suppressed during a UTI.

The typical pH range of healthy urine is pH 6.0–7.5, which is often lower than LB medium. Therefore, we hypothesized that the pH of the medium could affect mucoidy or extracellular polysaccharide (EPS) production and localization. To discriminate between cell-associated EPS (i.e., CPS) versus cell-free EPS, we quantified the total uronic acid content of EPS extracted from both bacteria and culture medium (total EPS) versus the spent culture supernatant (cell-free EPS). Cell-associated CPS was inferred by subtracting cell-free EPS from total EPS. This approach circumvented technical issues caused by poor sedimentation, which confounds direct measurement of cell-associated CPS as it is challenging to cleanly separate cells from the supernatant. We cultured KPPR1 in LB medium adjusted to pH 5, 6, 7, 8, and 9, then quantified mucoidy by sedimentation and EPS localization by uronic acid content (Fig. S2). We observed that pH 9 increased sedimentation resistance and cell-free EPS production, while pH 5 reduces sedimentation resistance and cell-free EPS production. However, these changes in sedimentation resistance were not as dramatic as what was observed in urine (Fig. 1A and B) and did not occur in the pH range between the pooled, human urine (pH 6.5) and LB medium (pH 7.2–7.5). These data indicated that basic pH increases both sedimentation resistance and cell-free EPS but that is not likely the factor by which urine suppresses mucoidy.

To examine CPS biosynthesis and mucoidy by another measure, we quantified the transcript abundance of each gene in the KPPR1 CPS biosynthetic gene cluster and *rmp* locus in urine relative to LB medium. CPS biosynthetic genes were not broadly suppressed in urine compared to LB medium, but the *rmp* locus was significantly suppressed (fold change range = 0.207–0.326) (Fig. 1D). In the CPS locus, eight genes (*galF, VK055_5018, VK055_5019, VK055_5023, VK055_5024, manC, manB,* and *ugd*) were significantly down-regulated although the fold change was minor (range = 0.704–0.850). Three genes (*orf2, wzi,* and *gnd*) were significantly up-regulated, again with a minor fold-change increase (range = 1.256–1.545). These fold changes do not impact the amount of cell-associated CPS produced by KPPR1, as measured by uronic acid content (Fig. 1B). These results indicate that urine may reduce the mucoid phenotype by down-regulating *rmpD* transcription.

### A screen for genes required for urine-suppressed mucoidy

To identify bacterial factors acting downstream of RmpD to suppress mucoidy in urine, we screened an arrayed KPPR1 transposon (Tn) library for mutants with increased sedimentation resistance in urine. The transposon library contains 3,733 mutants, which disrupt 72% of all open reading frames (ORFs) (19). The library was cultured in sterile, pooled human urine, and the sedimentation resistance of each mutant was assessed. With a hit rate of 2.4%, the 89 mutants initially identified to have increased sedimentation resistance were evaluated for mucoidy by string test on urine agar and sedimentation resistance in triplicate. Eighteen of 89 primary hits appeared mucoid either on urine agar or in the secondary sedimentation assay performed in triplicate. We noted that some of these 18 hits had mixed colony morphology which often matched observed changes in sedimentation resistance (Table 1). Therefore, the string test on urine agar was repeated for each isolate. Ultimately, 13 transposon mutants had at least one isolate that passed all screening and confirmation (Table 1). Individual isolates that passed all screens tended to appear shinier, more translucent, and exhibited less distinct colonies on solid growth medium (Fig. S3).

**TABLE 1:** Tn:mucoid^WT^ and Tn:mucoid^+^ isolate phenotypes and genotypes

Clone ID	Gene	Primary screen: sedimentation^*a*^	Secondary screen: string test^*b*^	Secondary screen: sedimentation^*c*^	Individual isolate: string test^*b*^	Tn:mucoid WT vs. +	WGS non-synonymous variants^*d*^	Wzc Sanger sequencing^*e*^	Wzc residue^*f*^
	*VK055_4579_pheA*	0.105	+	0.0573					
1 B1	*VK055_4579_pheA*			0.064	+	+	*VK055_5017_wzc_C1939T_*	*VK055_5017_wzc_C1939T_*	P646S
2 B1	*VK055_4579_pheA*			0.054	0	WT	*VK055_2438*_C397T_ *VK055_4590*_A34C_		
3 B1	*VK055_4579_pheA*			0.054	0	WT	None reported		
	*VK055_3922_yraO*	0.053	+	0.0577					
1 F2	*VK055_3922_yraO*			0.058	+	WT	–		
1 F2g **^*g*^**	*VK055_3922_yraO*			–	+	+	–	*VK055_5017_wzc_C1183A_*	Q395K
2 F2	*VK055_3922_yraO*			0.058	+	WT	–		
3 F2	*VK055_3922_yraO*			0.057	+	+	–	*VK055_5017_wzc_C1183A_*	Q395K
	*Tn insertion unknown*	0.192	+	0.089					
4 B4	*Tn insertion unknown*			0.076	+	+	*VK055_1978_bepE_T659G_*	*VK055_5017_wzc_G1709T_*	G569V
5 B4	*Tn insertion unknown*			0.137	+	+	*VK055_5017_wzc_G1709T_ VK055_0186_vgrG2_G2248A_ VK055_1734*_A212C_	*VK055_5017_wzc_WT_ VK055_5017_wzc_G1709T_*	WT G569V
6 B4	*Tn insertion unknown*			0.054	0	WT	None reported		
	*VK055_1828*	0.06	+	0.0587					
7 C8	*VK055_1828*			0.055	0	WT	*VK055_5031*_T406A_		
8 C8	*VK055_1828*			0.057	0	WT	*VK055_4839_1382ΔG_ intergenic_3A938201G_*		
9 C8	*VK055_1828*			0.064	+	+	*VK055_5017_wzc_G1709T_*	*VK055_5017_wzc_G1709A_*	G569D
	*VK055_2345*	0.08	+	0.0577					
7 C9	*VK055_2345*			0.056	+	+	–	*VK055_5017_wzc_C1183A_*	Q395K
8 C9	*VK055_2345*			0.061	+	+	–	*VK055_5017_wzc_C1183A_*	Q395K
9 C9	*VK055_2345*			0.056	+	+	–	*VK055_5017_wzc_C1183A_*	Q395K
	*VK055_4105*	0.061	+	0.1143					
7 C10	*VK055_4105*			0.099	+	+	–	*VK055_5017_wzc_G1708T_*	G569C
8 C10	*VK055_4105*			0.111	+	+	–	*VK055_5017_wzc_G1708T_*	G569C
9 C10	*VK055_4105*			0.133	+	+	–	*VK055_5017_wzc_G1708T_*	G569C
	*VK055_1812_dtpD*	0.077	+	0.096					
7 E2	*VK055_1812_dtpD*			0.139	+	+	–	*VK055_5017_wzc_WT_ VK055_5017_wzc_G1709A_*	WT G569D
8 E2	*VK055_1812_dtpD*			0.057	+	+	–	*VK055_5017_wzc_WT_ VK055_5017_wzc_G1709A_*	WT G569D
9 E2	*VK055_1812_dtpD*			0.092	+	+	–	*VK055_5017_wzc_G1708T_*	G569C
	*VK055_4261*	0.075	+	0.0553					
7 E3	*VK055_4261*			0.056	+	+	–	*VK055_5017_wzc_G1708A_*	G569D
8 E3	*VK055_4261*			0.055	+	+	–	*VK055_5017_wzc_G1708A_*	G569D
9 E3	*VK055_4261*			0.055	+	+	–	*VK055_5017_wzc_G1708A_*	G569D
	*VK055_0874*	0.064	+	0.0557					
7 E5	*VK055_0874*			0.055	+	+	–	*VK055_5017_wzc_G1708A_*	G569D
8 E5	*VK055_0874*			0.054	+	+	–	*VK055_5017_wzc_G1708A_*	G569D
9 E5	*VK055_0874*			0.058	+	+	–	*VK055_5017_wzc_G1708A_*	G569D
	*VK055_4849_nuoN*	0.054	+	0.0573					
7 F7	*VK055_4849_nuoN*			0.057	0	WT	*VK055_4892_mqo2_T1289G_* *VK055_0435* C587T		
8 F7	*VK055_4849_nuoN*			0.057	+	+	*VK055_5017_wzc_G1709T_*	*VK055_5017_wzc_G1709T_*	G569V
9 F7	*VK055_4849_nuoN*			0.058	+	+	None reported	*VK055_5017_wzc_G1709T_*	G569V
	*VK055_4923_pfkB*	0.059	+	0.0653					
7 F9	*VK055_4923_pfkB*			0.084	0	WT	None reported	*VK055_5017_wzc_T798C_*	WT
7 F9g	*VK055_4923_pfkB*			–	+	+	*VK055_5017_1576_insGCA_*	*VK055_5017_1576_insGCA_*	526_insA
8 F9	*VK055_4923_pfkB*			0.057	0	WT	*VK055_0184*_A1007T_		
9 F9	*VK055_4923_pfkB*			0.055	0	WT	None reported		
	*VK055_2280_proB*	0.065	+	0.069					
7 H8	*VK055_2280_proB*			0.056	0	WT	*VK055_3855_G211T_ intergenic_C879584A_*		
8 H8	*VK055_2280_proB*			0.056	0	WT	None reported		
9 H8	*VK055_2280_proB*			0.095	+	+	*VK055_5017_wzc_G1709T_* *VK055_1078*_G586T_	*VK055_5017_wzc_WT_ VK055_5017_wzc_G1709A_* *VK055_5017_wzc_G1709T_*	WT G569D G569V
	*VK055_1417*	0.069	+	0.0547					
10 D5	*VK055_1417*			0.055	0	WT	–		
10 D5g	*VK055_1417*			–	+	+	–	*VK055_5017_wzc_C1183A_*	Q395K
11 D5	*VK055_1417*			0.054	+	+	–	*VK055_5017_wzc_C1183A_*	Q395K
11 F10	*VK055_1417*			0.055	+	+	–	*VK055_5017_wzc_C1183A_*	Q395K

^*a*^ OD_600_ after sedimenting microplate at 2,000 × *g* for 20 min. Hits: ≥2 standard deviations from the plate mean.

^*b*^ 0 = no string; + = string > 5 mm.

^*c*^ OD_600_ after sedimenting 3 mL culture at 7,000 × *g* for 10 min. Hits: ≥2 standard deviations from wild-type KPPR1 mean.

^*d*^ Nucleotide changes detected by whole-genome sequencing.

^*e*^ Nucleotide changes in Wzc detected by Sanger sequencing.

^*f*^ Wzc amino acid residue change.

^*g*^ Clone IDs appended with a “g” were colony purified after the 3 mL sedimentation assay.

**Fig 2:**
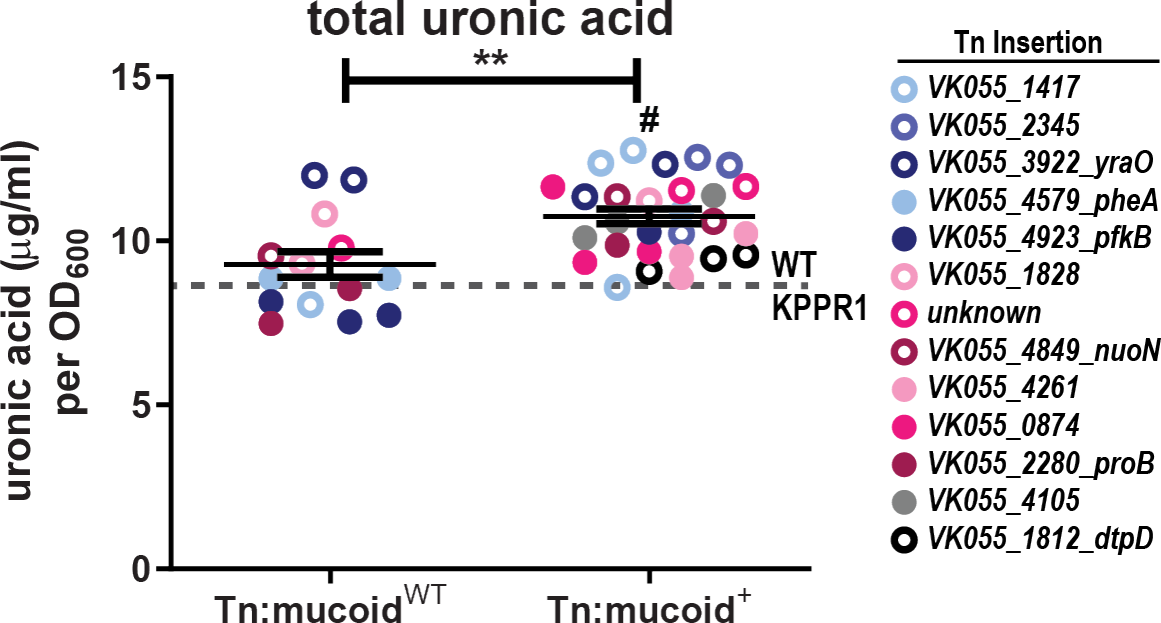
Transposon isolates with elevated mucoidy produce more extracellular uronic acid. Each *K. pneumoniae* transposon isolate (Tn:mucoid^WT^ or Tn:mucoid^+^) was cultured in triplicate in LB medium, where *N* = 42 isolates representing 13 distinct transposon insertion sites. Total extracellular polysaccharides (cell-free EPS and CPS combined) were extracted from total culture, and the uronic acid content was determined and normalized to the OD_600_ of the overnight culture. The legend on the right identifies the marker color that corresponds to each transposon insertion site. Each marker represents an individual isolate, and the gray dashed line represents averaged WT KPPR1 data (*N* = 24; mean = 8.717; SD = 1.082). A Student’s *t* test was used to determine if the mean extracellular uronic acid produced in strains that exhibit elevated mucoidy (Tn:mucoid^+^) was significantly different compared to transposon strains that exhibited wild-type mucoidy (Tn:mucoid^WT^; **P < 0.0021) or WT KPPR1 (^#^ *P* < 0.0001).

Elevated CPS production is associated with increased mucoidy, although there is strong evidence that other factors contribute to mucoidy (18, 19). Each of the 13 transposon mutants had three or four isolates that exhibited either wild-type (WT) (Tn:mucoid^WT^) or increased mucoidy (Tn:mucoid^+^) (*N* = 42 isolates total) (Table 1). On average, the Tn:mucoid^+^ isolates produce 1.16-fold more total extracellular uronic acid compared to Tn:mucoid^WT^ isolates (Fig. 2). This difference is modest but significant. Notably, the two Tn:mucoid^WT^ isolates that produced the most uronic acid have transposon insertions in *yraO*. All four *yraO* transposon mutant isolates exhibit elevated uronic acid production (mean = 11.89 µg/mL uronic acid per OD_600_) regardless of mucoidy level (Fig. 2, dark blue, open markers). It is also notable that most Tn:mucoid^+^ strains produce total extracellular uronic acid quantities comparable to Tn:mucoid^WT^ strains (Fig. 2), adding further evidence that increased secretion of extracellular uronic acid-containing polysaccharides is not the only factor driving *K. pneumoniae* mucoidy.

### Tn:mucoid^+^ strains contain secondary mutations in *wzc*

The variation in mucoidy and sedimentation resistance observed between isolates derived from individual parental transposon-insertion strains was unexpected. The phenotype of each isolate was stable after passaging in LB medium. The transposon insertion site of each isolate was verified by PCR with one primer that anneals to the transposon and one primer that anneals to the expected genomic region. Since each hit had the expected transposon insertion site but exhibited varying mucoidy, we hypothesized that a secondary mutation was driving the increased mucoidy. We submitted purified genomic DNA from 19 isolates (*N* = 8 Tn:mucoid^+^ and *N* = 11 Tn:mucoid^WT^), plus wild-type KPPR1, for whole-genome sequencing (Table 1).

Sequence variants in each strain were identified using the variation analysis pipeline on PATRICbrc using *K. pneumoniae* subsp. *pneumoniae* strain VK055 (RefSeq: NZ_CP009208.1) as the reference strain (35, 36). Comparisons between our laboratory KPPR1 and the published genome identified one non-synonymous (insG) mutation at position 1,610,890 in *VK055_1597* and a synonymous (C > T) mutation at position 2,880,686 in *VK055_2811*. These variants were disregarded in all transposon isolates. Twenty-four other variants were identified (*N* = 1 deletion, *N* = 1 insertion, *N* = 16 non-synonymous, *N* = 2 intergenic, and *N* = 4 synonymous). The one insertion and six non-synonymous mutations all mapped to *VK055_5017_etk* (*wzc*) and were associated with 6 of the 8 Tn:mucoid^+^ isolates. No other genetic variations were associated with more than one Tn:mucoid^+^ isolate. Six unique variations associated with Tn:mucoid^+^ isolates included, *VK055_0186_vgrG2_2248G>A_, VK055_1078586_586G>T_*, *VK055_1734212_212A>C_, VK055_1978_bepE_659T>G_, VK055_4651_pbpC_2033T>G_,* and *VK055_4651_pbpC_2045C>G_* (Table 1). The remaining 13 variants were associated with Tn:mucoid^WT^ isolates (*N* = 7 of 12); none of the Tn:mucoid^WT^ isolates had point mutations in *wzc*. Ninety percent of Tn:mucoid^+^ had at least one non-synonymous variant, while 58% of Tn:mucoid^WT^ had at least one non-synonymous variant (Fig. S4), suggesting that the selection of Tn:mucoid^+^ strains enriched the number of secondary point mutations.

Since only 19 of the 42 transposon isolates were submitted for whole-genome sequencing, the *wzc* gene of all 42 isolates was PCR amplified, and the open reading frame interrogated for point mutations using Sanger sequencing. All Tn:mucoid^+^ had non-synonymous mutations in *wzc*, including the two isolates that did not have *wzc* mutations identified by WGS. None of the Tn:mucoid^WT^ isolates had *wzc* mutations. In four instances, Tn:mucoid^+^ appeared to be a mixture of wild-type and a residue change at Wzc_G569_ based on chromatogram (5 B4, 7 E2, 8 E2, and 9 H8) (Table 1), indicating that the phenotype is dominant if the colonies are not fully purified. Twice, WGS called *wzc*_G1709A_ and Sanger sequencing called *wzc*_G1709T_ (9 C8 and 9 H8) (Table 1), which could be due to heterogeneity at this base pair. Finally, we revived bacteria from the original transposon library wells to determine if the Wzc variants arose prior to the transposon screen or were generated in response to urine. Wzc variants were detected in all parental transposon wells, except *VK055_4923_pfkB* (Wzc_526insA_). These data indicate that the *wzc* mutations were primarily present prior to executing the transposon screen, suggesting that the effect of urine suppressing mucoidy allowed these naturally occurring Wzc variants, which increase mucoidy, to be identified.

**Fig 3:**
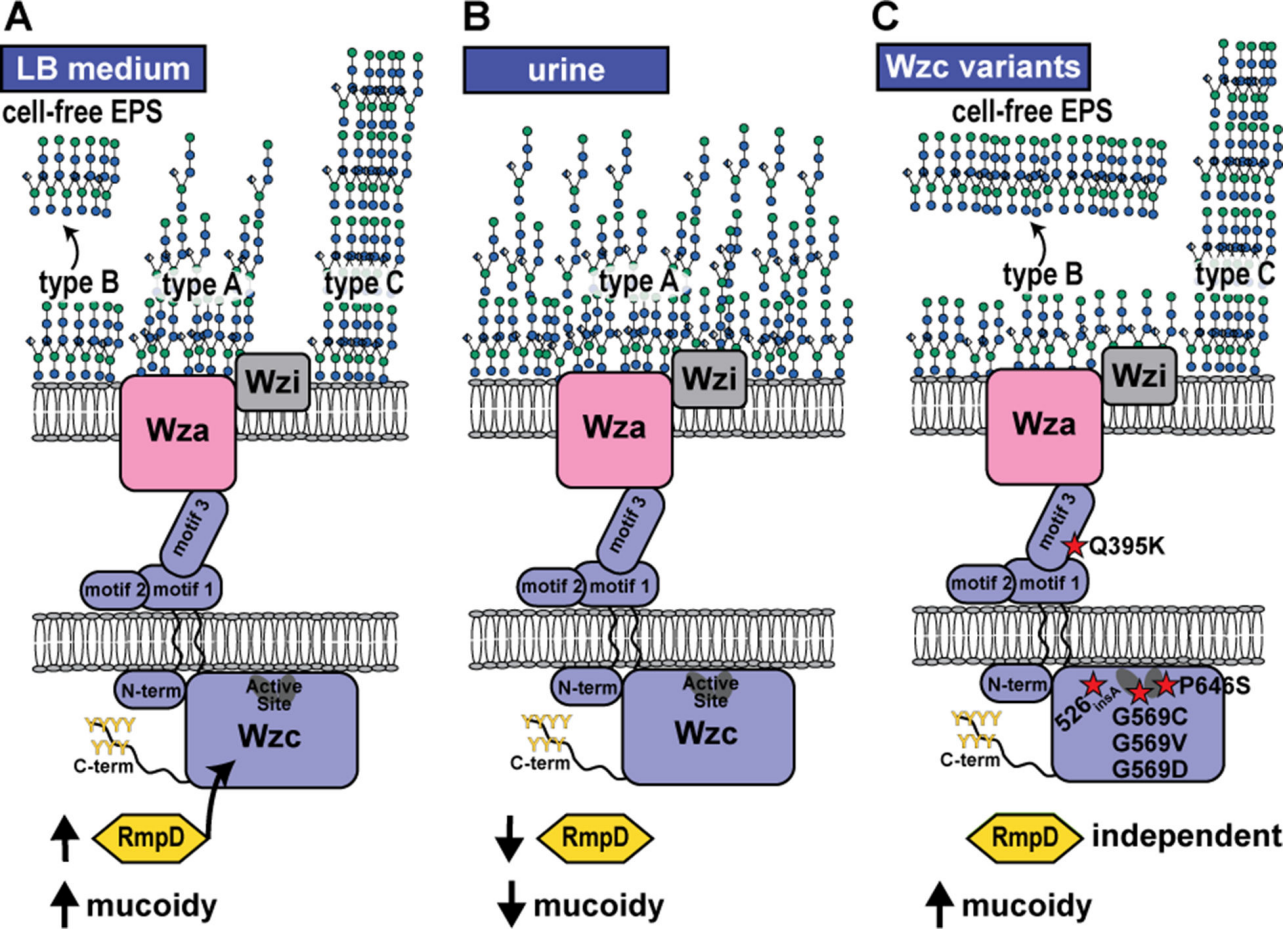
Model of *K. pneumoniae* control of capsule biosynthesis and mucoidy. Wzc is an inner membrane tyrosine kinase that regulates capsule (CPS) biosynthesis and extrusion. (**A**) When cultured in LB medium, *K. pneumoniae* KPPR1 presents as hypermucoid, which resists sedimentation. In this condition, three distinct cell-associated CPS species with different chain length properties are detected. Type A is a broad band with diverse chain lengths. Type B is a narrow band representing consistent chain lengths; this is the species also found as cell-free EPS. Type C is an ultra-high molecular weight CPS species. (**B**) Urine down-regulates *rmpD* transcription and results in lower mucoidy. Culturing *K. pneumoniae* in urine results in tightly cell-associated type A CPS production. (**C**) Six Wzc variants were identified here that overcome urine-induced mucoidy suppression independent of RmpD. The Wzc mutations are marked with a red star, where the dark ovals represent Walker A and B motifs. Wzc_Q395K_ is localized to the periplasmic motif 3, predicted to interact with the Wza outer membrane protein. Wzc_G569_ is adjacent to the active site tyrosine. Wzc_P646S_ is located in the active site Walker B motif. Wzc_526insA_ is located eight residues up-stream of the active site Walker A motif. All mutations result in constitutive production of type B CPS and copious secretion of type B CPS into the supernatant as cell-free EPS. All mutations, except Wzc_Q395K_, also increase production of cell-associate type C CPS, particularly in urine.

Six unique Wzc mutations were distributed across the Tn:mucoid^+^ strains. The Wzc mutations resulted in the following amino acid changes, Wzc_Q395K_ (*N* = 8), Wzc_526insA_ (*N* = 1), Wzc_G569C_ (*N* = 4), Wzc_G569V_ (*N* = 5), Wzc_G569D_ (*N* = 10), and Wzc_P646S_ (*N* = 1). Wzc is a conserved bacterial tyrosine kinase involved in CPS biosynthesis (21). It forms a periplasmic octamer that interfaces with the Wza octamer in the outer membrane and the Wzb phosphatase in the periplasm (22, 23, 37). The current model is that Wzc autophosphorylation dissociates the complex, and subsequent Wzb-mediated dephosphorylation results in complex re-assembly (23). It is these protein dynamics that drive CPS polymerization and Wza extrusion of nascent extracellular polysaccharides; loss of either Wzc phosphorylation or de-phosphorylation, as well as other mutations that alter Wzc molecular dynamics, disrupts CPS formation (22, 38). In this way, Wzc acts as a molecular timer that drives CPS biogenesis (38). Wzc is a two-membrane pass protein, where residues 1–30 and 448–722 are located in the cytoplasm and residues 51–427 are located in the periplasm. Only Wzc_Q395K_ is located in the periplasmic region in motif 3 (Fig. 3). Deleting motif 3 in structural studies of *E. coli* Wzc resulted in the loss of CPS production, with neither loss of octamerization nor loss of autokinase activity (23). Therefore, it is thought that motif 3 interfaces with Wza. The remaining point mutations are located in the C-terminal cytoplasmic region responsible for autokinase activity. Three Wzc_G569_ mutations were identified (G > C, G > V, and G > D) (Fig. 3). Wzc_G569_ is located on α4 and is adjacent to Wzc_Y570_, which is the active residue that autophosphorylates the poly-tyrosine C-terminus. Wzc_P646S_ is located in the Walker B motif involved in ATP binding in the active site (Fig. 3) (39). The alanine insertion after residue 526 is eight residues up-stream of a Walker A motif on the end of α2 (Fig. 3), a region shown to stabilize Mg^+2^-ATP binding (40).

### Tn:mucoid^+^ strains impact sedimentation resistance and increase cell-free EPS levels

**Fig 4:**
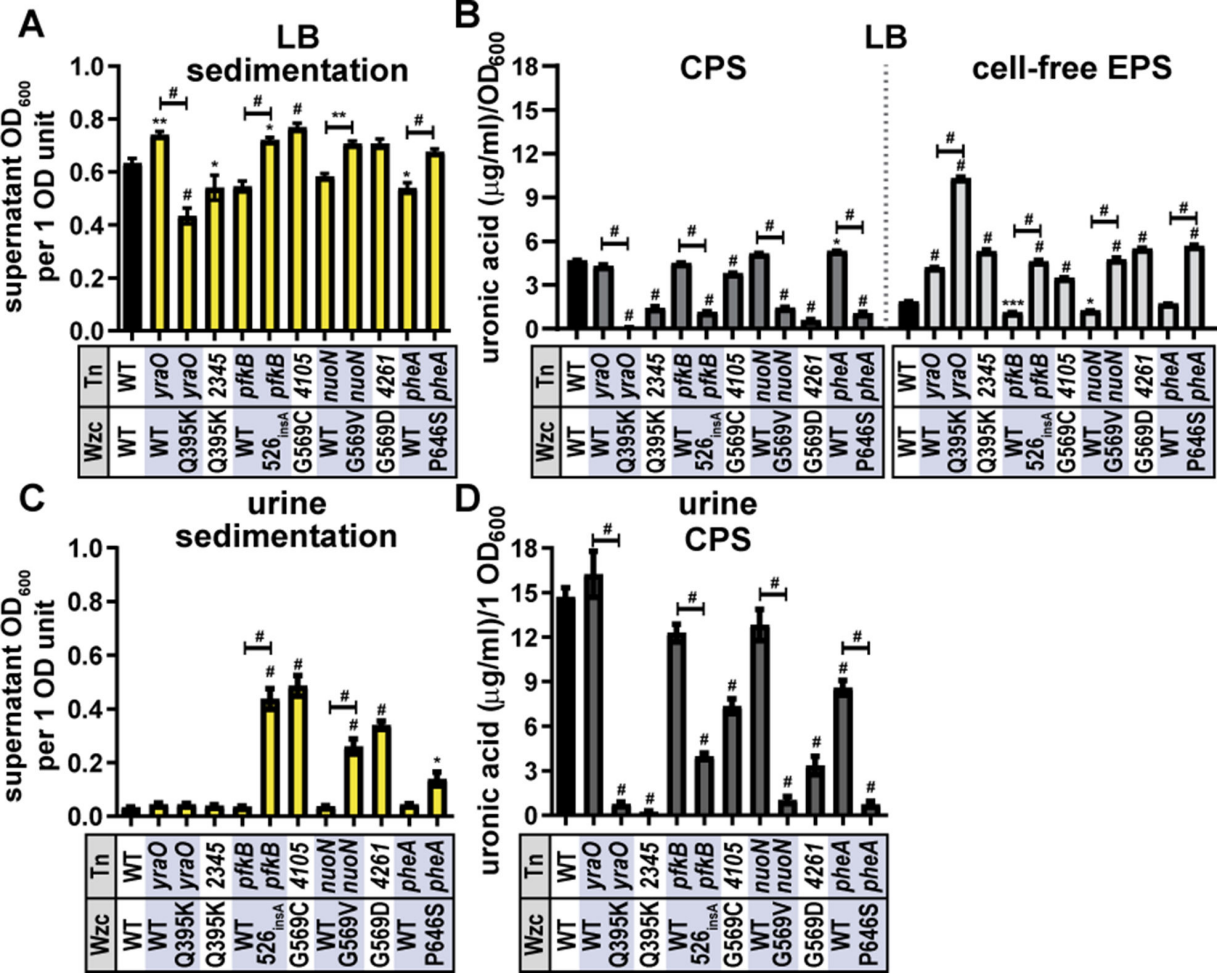
Tn:mucoid^+^ strains impact sedimentation resistance and EPS production. *K. pneumoniae* strain KPPR1 Tn:mucoid^+^ or Tn:mucoid^WT^ strains were cultured in (**A and B**) LB medium or (**C and D**) sterile-filtered human urine. The transposon (Tn) insertion site is listed on the X-axis along with the Wzc variant listed below (WT = wild type). (**A and C**) Mucoidy was determined by quantifying the supernatant OD_600_ after sedimenting 1 OD_600_ unit of culture at 1,000 × *g* for 5 min. (**B**) The uronic acid content of the total culture or supernatant (light gray, right) was quantified and normalized to the OD_600_ of the overnight culture. The cell-associated CPS (dark gray, left) was deduced by subtracting the cell-free uronic acid content (EPS) from the total culture uronic acid content. (**D**) Urine contains high levels of uronic acid-conjugated molecules. To eliminate this background signal, 1 OD_600_ unit of bacteria was washed in sterile PBS, and then the cell-associated uronic acid content was quantified. Data presented are the mean, and error bars represent the standard error of the mean. Statistical significance was determined using two-way ANOVA with a Bonferroni post-test to compare specific groups. *P < 0.0332; **P < 0.0021; ***P < 0.0002; ^#^ *P* < 0.0001. Experiments were performed ≥3 independent times, in triplicate.

We selected at least one Tn:mucoid^+^ isolate representative of each of the six Wzc point mutations. We prioritized mutants based on whether they had a partner Tn:mucoid^WT^ strain and if we had high confidence about their Wzc genotype based on sequencing results. All Tn:mucoid^+^ isolates resisted sedimentation in both LB and urine relative to wild-type KPPR1 and/or their Tn:mucoid^WT^ strain, except for Tn:mucoid^+^ strains encoding Wzc_Q395K_ (Fig. 4A and C). Similarly, the six Wzc mutations reduced cell-associated CPS and increased cell-free EPS production relative to wild-type KPPR1 and/or their Tn:mucoid^WT^ strain in LB medium (Fig. 4B). Urine contains high levels of uronidated molecules, which prohibit quantifying cell-free EPS. To quantify cell-associated CPS, 1 OD_600_ unit of bacteria cultured in urine was pelleted and washed with PBS prior to quantifying cell-associated uronic acid content. Again, the six Wzc mutations reduced cell-associated CPS relative to wild-type KPPR1 and/or their Tn:mucoid^WT^ strain (Fig. 4D). Since the Wzc_Q395K_ strains exhibited a different sedimentation resistance phenotype from the other Wzc mutant strains, we assayed two Tn:mucoid^+^ lineages, *yraO* (1 F2 and 1F2g) and *VK055_2345* (7 C9). Both isolates encoding Wzc_Q395K_ exhibited reduced sedimentation resistance despite overt tackiness of the colonies and viscosity of the cultures (Fig. 4). Furthermore, both Wzc_Q395K_ isolates primarily produce cell-free EPS, not cell-associated CPS. It is possible that the lack of cell-associated CPS in Wzc_Q395K_ causes these strains to appear visibly mucoid (viscous) but sediment well. Previous work has shown that some amount of cell-associated CPS is required for mucoidy to be apparent in the sedimentation assay (18, 19). To ensure that the dramatic changes in mucoidy and uronic acid production of *yraO* and *yraO-*Wzc_Q395K_ were not interfering with OD_600_ measurement, we verified that the OD_600_ of each strain is representative of a similar number of CFU/mL compared to KPPR1 (Fig. S1).

### Wzc mutations are sufficient to increase sedimentation resistance in wild-type KPPR1

**Fig 5:**
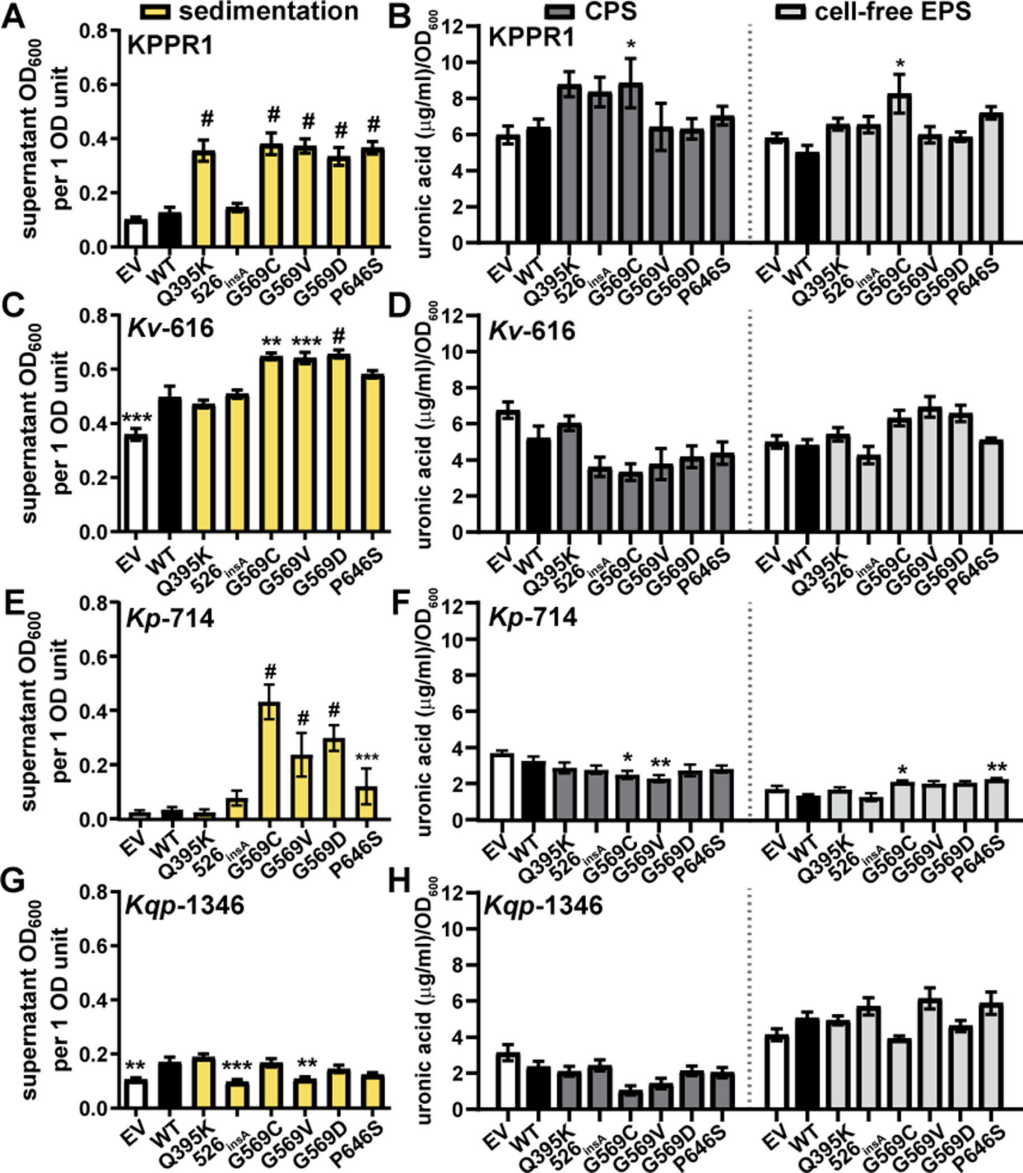
Inducing the expression of episomal Wzc variants with L-arabinose increases sedimentation resistance in hypervirulent and classical *Klebsiella* strains. Wild-type Wzc and six mutants were expressed with the pBAD18 arabinose-inducible promoter in wild-type KPPR1 (**A and B**), *K. variicola* (*Kv*) 616 (**C and D**), *K. pneumoniae* (*Kp*) 714 (**E and F**), or *K. quasipneumoniae* (*Kqp*) 1346 (**G and H**). Empty vector (EV) was used as a negative control. The strains were cultured in LB medium containing 50 mM L-arabinose and kanamycin. (**A, C, E and G**) Mucoidy was determined by quantifying the supernatant OD_600_ after sedimenting 1 OD_600_ unit of culture at 1,000 *× g* for 5 min. (**B, D, F and H**) Uronic acid content of the total culture or supernatant was quantified and normalized to the OD_600_ of the overnight culture. The cell-associated CPS was deduced by subtracting the cell-free uronic acid content (EPS) from the total culture uronic acid content. Data presented are the mean, and error bars represent the standard error of the mean. Statistical significance was determined using two-way ANOVA with a Bonferroni post-test to compare specific groups. *P < 0.0332; **P < 0.021; ***P < 0.0002; ^#^ *P* < 0.0001. Experiments were performed ≥3 independent times, in triplicate.

Since it became clear that the transposon insertion sites may also impact sedimentation resistance and CPS production (e.g*.*, *yraO*) (Fig. 4), we developed a system to discretely examine Wzc variant behavior. Loss of *wzc* results in periplasmic accumulation of CPS intermediates. Previous work has shown that similar *wza* and *wzy* mutants that accumulate CPS in the periplasm have broad cell envelope defects in addition to CPS loss (41). Therefore, we left the native *wzc* locus intact and introduced the Wzc variants on a pBAD18 plasmid under an arabinose-inducible promoter. The concentration of L-arabinose (50 mM) used in these experiments does not affect KPPR1 growth (Fig. S5). Except for Wzc_526insA_, over-expressing each Wzc variant significantly increased sedimentation resistance compared to over-expressing wild-type Wzc (range = 2.6- to 3.0-fold) (Fig. 5A). This indicates that the altered activity of variant Wzc proteins affects sedimentation resistance, even with an endogenous copy of wild-type Wzc. However, most point mutations did not increase CPS or cell-free EPS production, except for Wzc_G569C_ modestly increasing CPS (1.4-fold) and cell-free EPS (1.6-fold) (Fig. 5B). This suggests that the increased sedimentation resistance is not caused by cell-free EPS but some other effect of the Wzc variant proteins.

**Fig 6:**
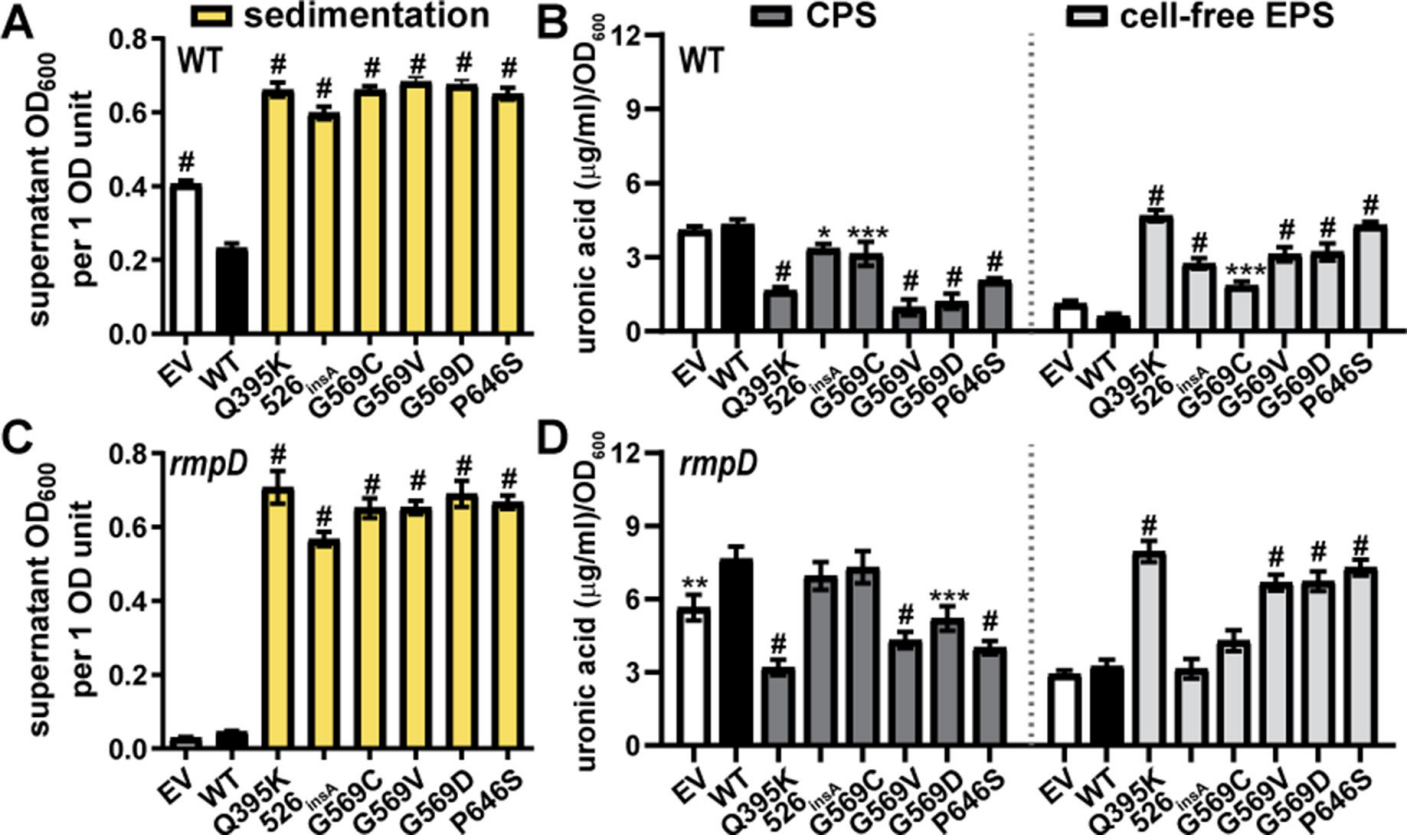
Episomal Wzc variants under native promoter control increase sedimentation resistance in KPPR1 wild type and *rmpD*. Wild-type Wzc and six mutants were expressed with the *wzi* promoter from pACYC184 in KPPR1 (**A and B**) WT and (**C and D**) *rmpD*. Empty vector (EV) was used as a negative control. The strains were cultured in LB medium. (**A and C**) Mucoidy was determined by quantifying the supernatant OD_600_ after sedimenting 1 OD_600_ unit of culture at 1,000 × *g* for 5 min. (**B and D**) Uronic acid content of the total culture or supernatant was quantified and normalized to the OD_600_ of the overnight culture. The cell-associated CPS was deduced by subtracting the cell-free uronic acid content (EPS) from the total culture uronic acid content. Data presented are the mean, and error bars represent the standard error of the mean. Statistical significance was determined using two-way ANOVA with a Bonferroni post-test to compare specific groups. *P < 0.0332; **P < 0.021; ***P < 0.0002; ^#^ *P* < 0.0001. Experiments were performed ≥3 independent times, in triplicate.

To ensure the rigor of these key results, wild type and the six Wzc variants were expressed *in trans* under the native *wzi* promoter using pACYC184∆*tet* in wild-type KPPR1 (19). Episomal expression of all six Wzc variants under the control of the native promoter also significantly increased sedimentation resistance compared to wild-type Wzc (range = 2.6- to 2.9-fold) (Fig. 6A). Furthermore, similar to the Tn:mucoid^+^ isolates (Fig. 4B), all six Wzc variants significantly reduced cell-associated CPS (range = 0.22- to 0.012-fold) and increased cell-free EPS compared to wild-type Wzc (range = 3.0- to 7.6-fold) (Fig. 6B).

### Wzc mutations are sufficient to increase sedimentation resistance in some *rmpD*-negative clinical UTI isolates

We then examined if the defined Wzc mutations are sufficient to increase sedimentation resistance in three clinical UTI isolates, which do not encode RmpD (Fig. 5C through J). pBAD18 vectors carrying wild-type *wzc* or the six variants were transformed into 616 (*K. variicola*), 714 (*K. pneumoniae*), and 1346 (*K. quasipneumoniae*). We were surprised to observe that over-expressing Wzc_G569C_, Wzc_G569V_, and Wzc_G569D_ *in trans* is sufficient to increase sedimentation resistance in *Kv-*616 and *Kp-*714; Wzc_P646S_ is also sufficient to increase sedimentation resistance in *Kp-*714 (Fig. 5C and E). In *Kv*-616, the Wzc variant-driven increase in sedimentation resistance was not associated with significant changes in CPS or cell-free EPS uronic acid content (Fig. 5D). Some of the Wzc variant-driven increases in sedimentation resistance in *Kp-*714 correlated with small decreases in CPS or small increases in cell-free EPS (Fig. 5F). Wzc variants did not significantly boost sedimentation resistance or EPS production in strain 1346 (Fig. 5I through J).

We translated the Wzc ORF from each strain and aligned the Wzc amino acid sequences from KPPR1, 616, 714, and 1346 using Clustal O (Fig. S6). Note that we were unable to retrieve the first 76 residues in the 714 Wzc sequence, likely due to a gap in sequencing coverage. All three strains encoded the wild-type residue for each tested variant (i.e., Q395, E525, G569, and P646). The pairwise identities between KPPR1 Wzc and each strain were as follows: 616 = 62.43%, 714 = 63.81%, and 1346 = 51.24%. We speculate that the native Wzc sequence or related interfacing proteins also contribute to whether a Wzc variant will induce mucoidy, but at this time, it is unclear what those sequence determinants are.

### Wzc mutations are sufficient to increase sedimentation resistance independent of RmpD in KPPR1

Since some Wzc variants were sufficient to increase mucoidy in classical strains lacking the *rmp* locus, we hypothesized that they may also be sufficient to increase mucoidy in KPPR1 ∆*rmpD*. Since ∆*rmpD* has a kanamycin-resistance marker, the chloramphenicol-resistant pACYC184-derived P*wzi-wzc* constructs were used. Episomal expression of all six Wzc variants under the control of the native promoter significantly increased sedimentation resistance compared to wild-type Wzc (range = 12.4- to 15.5-fold) (Fig. 6C). Furthermore, four Wzc variants significantly reduced cell-associated CPS (range = 0.42- to 0.68-fold) and increased cell-free EPS compared to wild-type Wzc (range = 2.1- to 2.6-fold) (Fig. 6D). Together, the data in Fig. 5 and 6 demonstrate that specific Wzc variants are sufficient to increase sedimentation resistance regardless of if a strain encodes *rmpD* or not.

### Wzc phosphorylation state is altered by some Wzc variants but not in urine

Wzc possesses autokinase function and coordinates CPS polymerization and extrusion. Since the Wzc variants function downstream of RmpD activity to control *Klebsiella* mucoidy, we next evaluated the effect of urine and the identified Wzc variants on these three activities. We hypothesized that Wzc variants oppose the effect of urine on Wzc phospho-status, CPS polymerization, and/or CPS secretion.

**Fig 7:**
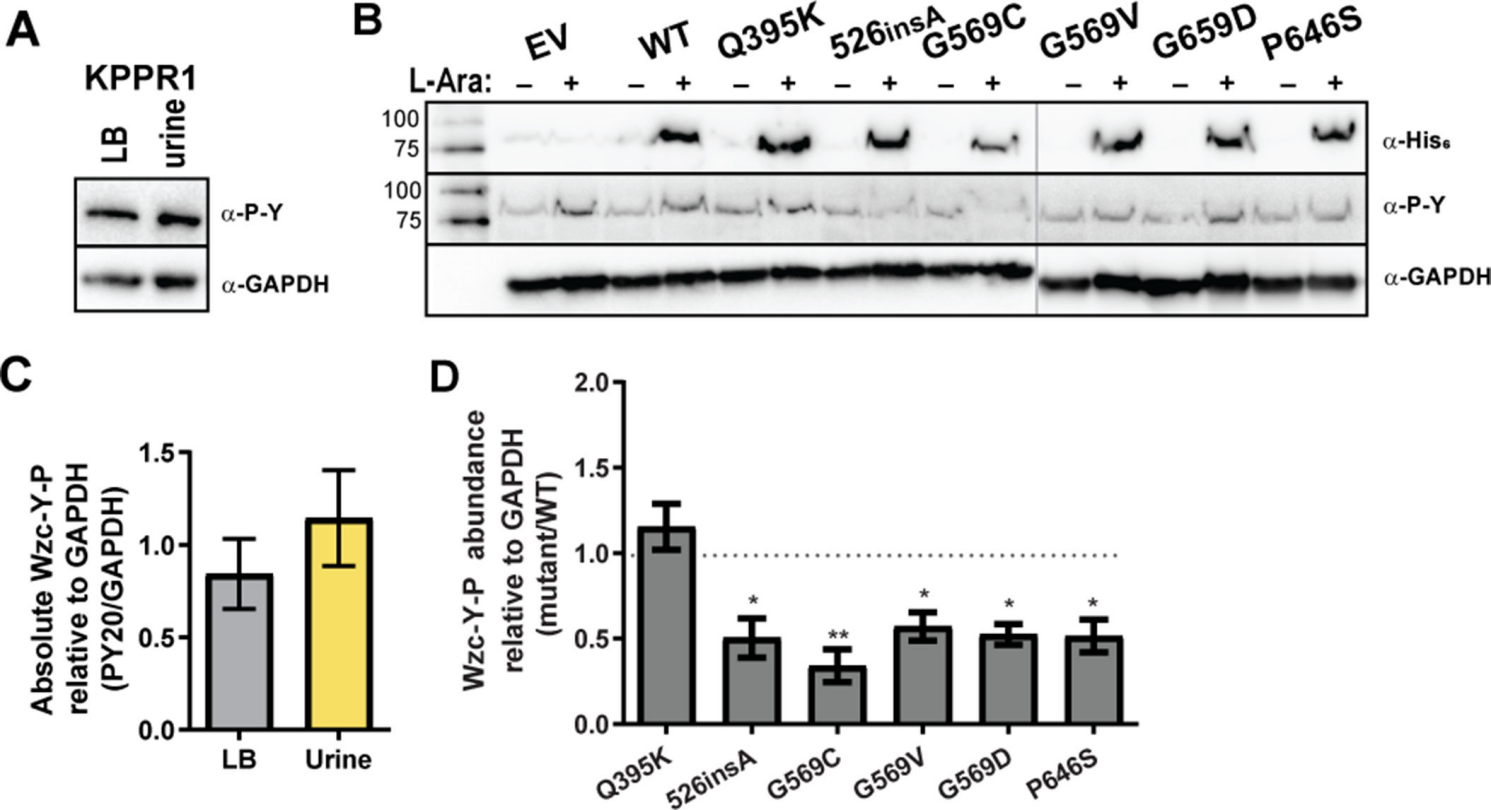
Wzc phosphorylation status in urine and with Wzc variant expression. (**A**) Wild-type KPPR1 was cultured in LB medium or sterile, pooled human urine. Whole cell lysates were resolved by SDS-PAGE, transferred to nitrocellulose, and then probed with anti-phosphotyrosine antibody (α-P-Y, PY20). Membranes were stripped and re-probed with anti-GAPDH (α-GAPDH, GA1R). (**B**) KPPR1 transformed with His_6_-tagged Wzc (WT, Q395K, 526_insA_, G569C, G569V, G569D, P646S), or empty vector (EV) pBAD18 was cultured in LB medium containing kanamycin with (+) and without (−) 50 mM L-arabinose. The whole cell lysates were similarly analyzed by western blot using α-P-Y, anti-His_6_ (α-His_6_, SB194b), or α-GAPDH antibodies, as in panel **A**. The vertical gray line demarks samples run on different gels. (**C**) The Wzc-Y-P bands in panel **A** were quantified in ImageJ and normalized to GAPDH band intensity. No significant difference was detected by an unpaired *t*-test. (**D**) The Wzc-Y-P bands in panel **B** were quantified in ImageJ and normalized to GAPDH band intensity. Normalized Wzc-Y-P in each mutant was divided by the normalized Wzc-Y-P of Wzc^WT^ within each blot. A one-way ANOVA with a Bonferroni post-test was used to determine if each mean was significantly different from 1.0 (WT), where *P < 0.0332; **P < 0.0021; ***P < 0.0002. Representative images are shown in panels **A and B, **and data presented in panels **C and D** represent the mean of ≥3 independent experiments with error bars representing the standard error of the mean.

To examine the effect of urine or the identified Wzc variants on Wzc phosphorylation status, whole-cell lysates were separated by SDS-PAGE and their phospho-tyrosine (Y-P) profiles were probed by western blot. As expected, we observed Wzc Y-P at 80 kDa with anti-phosphotyrosine antibody (PY20) (Fig. 7A and B). The 80 kDa band was confirmed to be Wzc Y-P by inducing expression of Wzc-His_6_ (C-terminal) with L-arabinose; anti-His_6_ antibodies detected Wzc-His_6_ at 80 kDa (Fig. 7B). We observed that Wzc-Y-P abundance is unchanged when KPPR1 is cultured in LB medium versus sterile-filtered urine (Fig. 7A and C). We then probed Wzc Y-P abundance wild-type KPPR1 carrying pBAD18 empty vector (EV), WT, and each of the six Wzc mutations with PY20. All Wzc variants decrease Wzc Y-P abundance compared to Wzc_WT_, except Wzc_Q395K_ (periplasmic) Y-P abundance remains similar to Wzc_WT_ (Fig. 7B and D). All pBAD18 vectors over-expressing Wzc-His_6_ variants exhibited increased sedimentation resistance compared to wild type, except for Wzc_526insA_ (Fig. S7). The Tn:mucoid^WT^ and Tn:mucoid^+^ isolates were also probed for changes in Wzc phosphorylation status, although we were unable to monitor total Wzc levels as the Wzc protein is not His-tagged in these strains (Fig. S8). In this genetic system with chromosomal Wzc variants, the *yraO-*Wzc_Q395K_ isolate was the only strain that significantly increased Wzc Y-P abundance; however, all G569 variants trended toward reduced Wzc Y-P abundance (Fig. S8). Recall that despite appearing mucoid, *yraO-*Wzc_Q395K_ exhibited reduced sedimentation resistance and distributed all EPS into the culture supernatant (Fig. 4A and B). All transposon strains encoding Wzc_G569_ variants also appeared mucoid and exhibited increased sedimentation resistance; although they released most EPS into the culture supernatant, some were retained as cell-associated CPS (Fig. 4A and B).

### Wzc variants overcome urine-induced changes in extracellular polysaccharide chain length and localization

To examine the effect of urine or the identified Wzc variants on extracellular polysaccharide localization and chain length, purified extracellular polysaccharides were visualized using gel electrophoresis and polysaccharide staining. The strains interrogated included wild-type KPPR1 and all Tn:mucoid^+^ strains with cognate Tn:mucoid^WT^ strains. Cell-associated CPS and cell-free EPS were isolated from each strain after they were cultured in LB or urine. The purified cell-associated CPS and cell-free EPS samples were separated by SDS-PAGE and visualized using alcian blue then silver stain.

**Fig 8:**
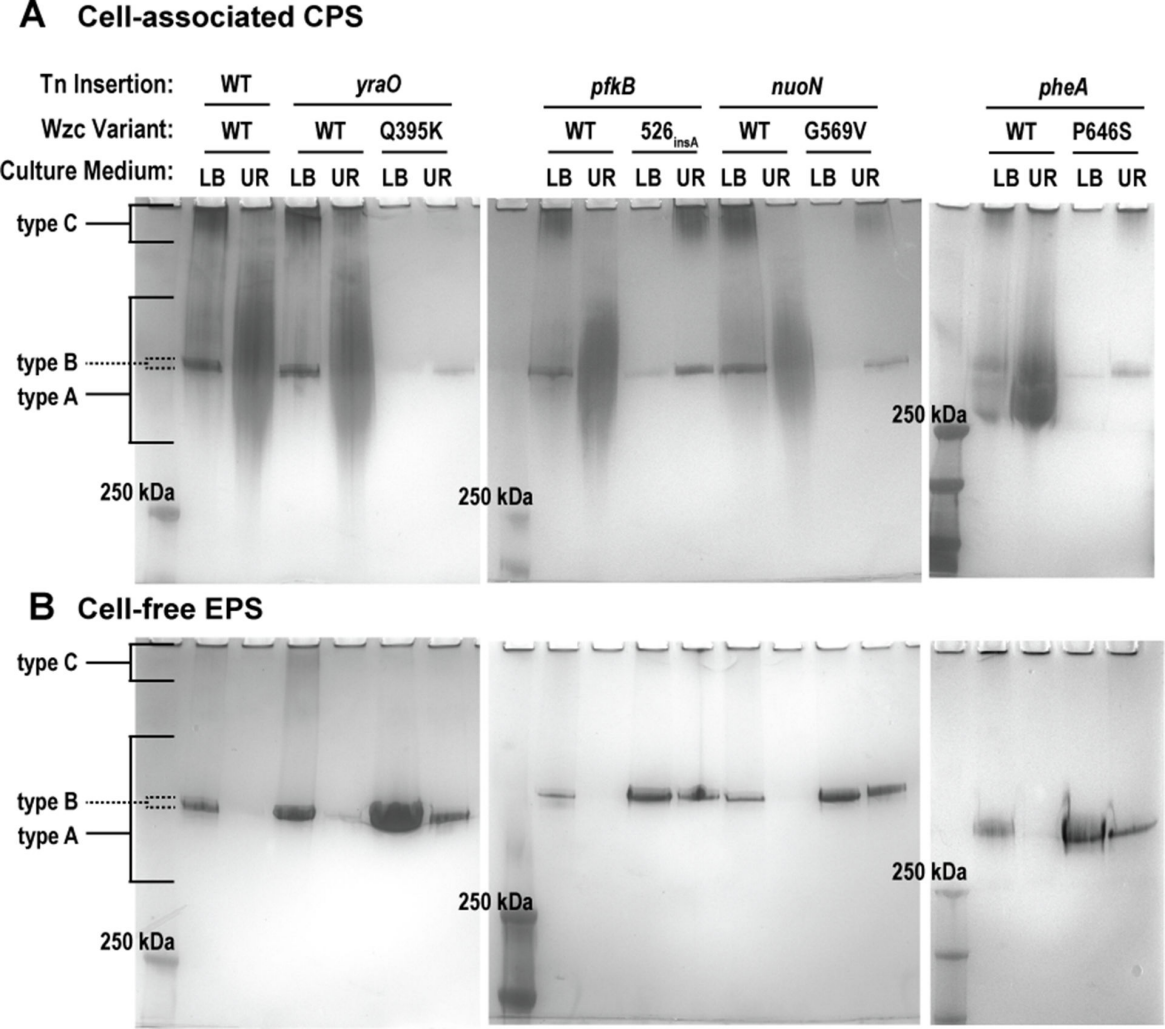
Cell-associated and cell-free extracellular polysaccharide profiles in urine and Wzc variants. Wild-type (WT) *K. pneumoniae* strain KPPR1 or derived Tn:mucoid^WT^ and Tn:mucoid^+^ strains were cultured in LB medium or sterile-filtered human urine (UR). The Tn insertion site and Wzc variant are reported above each lane. (**A**) Cell-associated capsule (CPS) and (**B**) cell-free extracellular polysaccharide (EPS) were purified from bacterial strains cultured in each condition and resolved on a 4%–15% SDS-PAGE gel. Polysaccharides were stained with alcian blue and then enhanced with a silver stain. Three distinct polysaccharide species were detected: diverse, mid- to high-molecular-weight chains (type A); uniform, high-molecular-weight polysaccharide chains (type A); and diffuse, ultra-high-molecular-weight chains (type C). Representative images are shown of ≥3 independent experiments.

When cultured in LB medium, wild-type KPPR1 cell-associated CPS has three distinct polysaccharide bands, representing three types of polysaccharide chain lengths. The “type A” polysaccharides represent diverse mid- to high-molecular-weight chains. The “type B” polysaccharides represent uniform, high-molecular-weight polysaccharide chains. The “type C” polysaccharides represent diffuse ultra-high-molecular-weight chains, retained at the top of the gel (Fig. 8A). When wild-type KPPR1 is cultured in urine, the uniform type B polysaccharide chains are absent from the CPS; instead, the CPS appears to be composed of primarily diverse type A polysaccharide, along with some ultra-high-molecular-weight type C polysaccharide (Fig. 8A). These chain length changes observed in LB medium versus urine are recapitulated in all Tn:mucoid^WT^ strains (Fig. 8A). However, when cultured in LB medium, all Tn:mucoid^+^ strains lose the diverse type A polysaccharide chains and primarily produce the uniform band type B polysaccharides in their capsules (Fig. 8A). Unlike wild-type KPPR1 or Tn:mucoid^WT^ strains, all Tn:mucoid^+^ strains resist urine-induced production of diverse type A polysaccharide chains and continue to produce the narrow type B capsular polysaccharides in urine (Fig. 8A). Furthermore, culturing the Tn:mucoid^+^ isolates with Wzc_526insA_, Wzc_G569V_, and Wzc_P646S_ variants in urine enhanced production of the ultra-high-molecular-weight type C capsular polysaccharides, but this is not observed in the Tn:mucoid^+^ Wzc_Q395K_ isolate (Fig. 8A).

We then examined the properties of cell-free EPS isolated from these same strains cultured in LB medium or urine. Neither the diverse type A nor the ultra-high-molecular-weight type C polysaccharides are released into the culture supernatant in any strains when cultured in LB medium or urine, indicating that type A and C polysaccharides are tightly associated with the bacterial cell surface (Fig. 8B). Therefore, when cultured in LB medium, the cell-free EPS isolated from wild-type KPPR1 or Tn:mucoid^WT^ strains comprised uniform type B polysaccharide chains (Fig. 8B). However, when wild-type KPPR1 or the Tn:mucoid^WT^ strains are cultured in urine, all cell-free EPS is absent (Fig. 8B). This loss of type B polysaccharides from the cell-free EPS is not observed in the Tn:mucoid^+^ strains. All Tn:mucoid^+^ strains produce uniform type B polysaccharide chains in cell-free EPS isolated from both LB medium and urine, although the abundance is diminished in urine compared to LB (Fig. 8B). In both LB medium and urine, the release of the uniform type B polysaccharides into the culture supernatant is increased in Tn:mucoid^+^ compared to Tn:mucoid^WT^ strains.

Combined, these data support a model where urine coordinately increases *K. pneumoniae* cell-associated CPS type A chains, diminishes production of type B and C polysaccharide chains, and increases CPS association with the cell surface (Fig. 3A and B). The Wzc variants, identified to overcome urine-mediated suppression of sedimentation resistance, circumvent these effects of urine by shifting almost all synthesized extracellular polysaccharides to the uniform type B chain length and reducing attachment to the cell surface (Fig. 3C). Notably, Wzc_526insA_, Wzc_G569V_, and Wzc_P646S_ variants enhance production of the ultra-high-molecular-weight type C capsular polysaccharide in urine, but the Wzc_Q395K_ variant does not. Although Wzc_Q395K_, Wzc_526insA_, Wzc_G569V_, and Wzc_P646S_ variants all appear visibly mucoid on solid growth medium, the Wzc_Q395K_ variant does not exhibit increased sedimentation resistance like these other isolates (Fig. 4). Finally, in Fig. 5, we observed increased sedimentation resistance in some strains that did not produce increased cell-free EPS production. Therefore, we posit that the mucoid phenotype is primarily driven by the cell-associated ultra-high-molecular-weight type C polysaccharide chains present in *K. pneumoniae* CPS.

### Tn:mucoid^+^ strains evade association with bone marrow-derived macrophages

**Fig 9:**
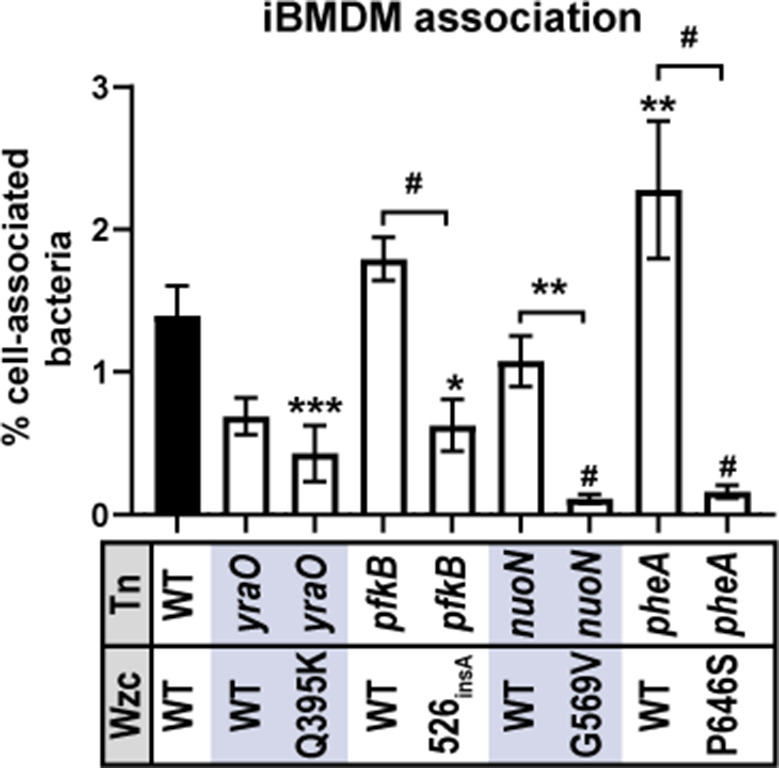
Tn:mucoid^+^ strains block bacterial association with immortalized bone marrow-derived macrophages (iBMDM). The ability of wild-type (WT) *K. pneumoniae* strain KPPR1 or derived Tn:mucoid^WT^ and Tn:mucoid^+^ strains to associate with iBMDM cells from *Mus musculus* was evaluated. Data presented are the mean, and error bars represent the standard error of the mean. Statistical significance was determined using two-way ANOVA with a Bonferroni post-test to compare specific groups. *P < 0.0332; **P < 0.021; ***P < 0.0002; ^#^ *P* < 0.0001. Experiments were performed ≥3 independent times, in triplicate.

To examine how altered CPS chain length and sedimentation resistance could affect interactions with the host, we quantified how well Tn:mucoid^WT^ versus Tn:mucoid^+^ strains were bound by immortalized bone marrow-derived macrophages from *Mus musculus* (iBMDMs, BEI #NR-9456). Generally, we found that Tn:mucoid^+^ bacteria did not associate as well with iBMDMs compared to wild-type KPPR1 or their Tn:mucoid^WT^ counterpart (Fig. 9). These data indicate that acquiring Wzc variants, which increase sedimentation resistance, primarily produce “type B” polysaccharide chains, and reduce cell-surface CPS attachment, can reduce bacterial binding and internalization by host immune cells.

## DISCUSSION

Here, we have found that *K. pneumoniae* species complex strains, including some that do not encode *rmpADC*, are less mucoid when cultured in human urine versus LB medium (Fig. 1A). However, CPS abundance is sustained or enhanced in urine (Fig. 1B). Recent work has shown that RmpD boosts mucoidy by controlling CPS chain length via Wzc interaction (18, 20). We observed that urine down-regulates the *rmpADC* locus compared to LB medium (Fig. 1D), suggesting that the effect of urine on mucoidy is likely due to decreased RmpD activity. We then screened a transposon library to identify genetic factors that could overcome urine-mediated suppression of mucoidy. Perplexingly, isolates with the same transposon insertion site exhibited different mucoid phenotypes. Whole-genome sequencing revealed that every isolate with increased mucoidy had a secondary mutation in the CPS biosynthetic gene, *wzc*. The six Wzc variants identified were located in the periplasm (Q395K), cytoplasm (526insA), and active site (G569C, G569V, G569D, and P646S) (Fig. 3).

With some exceptions, these Wzc variants increased sedimentation resistance in LB medium and urine and increased cell-free EPS (Fig. 4). We were unable to make targeted *wzc* knock-out or point mutant strains without observing secondary mutations. Similar challenges with genetically manipulating this ORF have been reported by other groups (26, 42). Therefore, we validated our findings using isogenic transposon mutant pairs with Wzc wild type versus variant alleles plus two plasmid-based systems: (i) arabinose induction from pBAD18 and (ii) native promoter control from pACYC184. Each system has its limitations, but when considered in combination, they provide compelling evidence that the identified Wzc variants counteract urine-mediated mucoidy suppression by regulating CPS chain length independent of RmpD.

Certain Wzc variants were sufficient to increase mucoidy in wild-type KPPR1 (encoding *rmpD*) and two classical *Klebsiella* UTI isolates (lacking *rmpD*) when expression was induced with L-arabinose from the pBAD18 plasmid (Fig. 5). Increased sedimentation resistance due to pBAD18 over-expression of Wzc variants did not increase cell-free EPS in KPPR1 or 616 (Fig. 5). Furthermore, all Wzc variants were sufficient to increase sedimentation resistance in wild-type and ∆*rmpD* KPPR1 when expressed under the native promoter on pACYC184 (Fig. 6). Together, these data indicate that naturally acquired Wzc variants can increase mucoidy independently of *rmpD* in both hypervirulent and classical *Klebsiella* strains.

Although urine and the Q395K variant do not affect Wzc phospho-status, the four active site mutations all decreased Wzc phospho-status; 526_insA_ only decreased Wzc phosopho-status when expressed from pBAD18 (Fig. 7; Fig. S8). Notably, in comparison to LB medium, culturing wild-type KPPR1 in urine dramatically increased cell-associated CPS chain length diversity and inhibited the release of EPS into the supernatant (Fig. 3 and 8). All Wzc variants induced constitutive production of a uniform “type B” polysaccharide in both LB medium and urine. Moreover, all variants enhanced the release of “type B” polysaccharides into the culture supernatant, even in urine (Fig. 3 and 8). All Wzc variants, except Q395K, also enhanced production of a cell-associated ultra-high-molecular “type C” polysaccharide, particularly in urine (Fig. 3 and 8). All Wzc variants reduced the ability of iBMDMs to bind and internalize the bacteria (Fig. 9).

In the context of a UTI, we propose that our data support the following working model. Upon entering the bladder, the signals present in urine suppress *rmpD* expression, increasing CPS chain length diversity and cell-surface attachment by directly modulating Wzc activity. How context-dependent control of CPS attachment versus release impact *K. pneumoniae* fitness remains undefined. Analogous regulated release of CPS in response to the host has been reported for *Streptococcus pneumoniae* (43, 44). Based on published data, non-mucoid *K. pneumoniae* adhere better to host cells, including bladder epithelial cells (19, 26). This is likely due to increased exposure of type 1 (*fim*) and type 3 (*mrk*) fimbriae on the bacterial surface, which are critical for establishing *K. pneumoniae* UTI (45 – 49). We propose that binding of *K. pneumoniae* to bladder epithelial cells is critical for establishing and persisting during UTI, akin to uropathogenic *E. coli* (50, 51). However, increased mucoidy blocks binding and internalization by host immune cells, such as macrophages. Therefore, we posit that spontaneous Wzc variants that emerge during infection could represent an invasive sub-population capable of resisting host defenses in the bloodstream or organs (52). This is supported by evidence that *rmpD-*negative ST258 isolates from human bloodstream infections have been identified, which exhibit increased mucoidy, encode Wzc variants, and escape phagocytosis (26).

To the best of our knowledge, this is the first example of *K. pneumoniae* modulating CPS chain length in response to human cues. The precise input signals controlling CPS chain length and cell-surface attachment remain to be defined. We hypothesize that a variety of signals may be integrated by the bacteria to fine-tune extracellular polysaccharide chain length and localization, thus improving fitness in distinct niches within the host or environment. Previous studies have demonstrated that *Acinetobacter baumannii* regulates EPS abundance and mucoidy in response to antibiotics and that *Streptococcus pneumoniae* regulates CPS chain length in response to polysaccharide pre-cursors, UDP-Glc and UDP-GlcA (42, 53). In *K. pneumoniae,* others have recently reported that L-fucose boosts sedimentation resistance independent of CPS abundance, and we have noted here that L-arabinose suppresses sedimentation resistance independent of CPS abundance, although the effects of these conditions on CPS chain length and attachment have not yet been determined (54). Therefore, it is possible that multiple exogenous signals could modulate intracellular levels of CPS pre-cursors to regulate CPS chain length in *K. pneumoniae*.

Data presented here, however, demonstrate an alternative route for *K. pneumoniae* to alter surface-exposed polysaccharide chain length and localization. It seems that a sub-population encoding Wzc variants supports constitutive *K. pneumoniae* mucoidy regardless of environmental cues. A few studies have identified Wzc mutations that increase mucoidy in *K. pneumoniae* and *A. baumannii* (26, 42, 55, 56). Wzc mutations reported in *A. baumannii* increased mucoidy, increased production of higher molecular weight EPS, and were typically associated with reduced Wzc-Y-P abundance (42). However, some of the reported mutants did not reduce Wzc-Y-P, akin to what we have observed with Wzc_Q395K_ and Wzc_526insA_ in *K. pneumoniae* (Fig. 7). Other studies have identified Wzc variants in *K. pneumoniae,* but none examined the impact on tyrosine phosphorylation. One study did observe a similar shift to what we call “type B” polysaccharide production in a primary ST11 clinical isolate with a C-terminal Wzc mutation (56), while another did not observe this shift in KPPR1S (20). We believe that the latter study did not observe enhanced type B polysaccharide in KPPR1S possibly due to examining combined CPS and EPS, along with high levels of RmpD expression in the experimental conditions and co-expression of Wzc_WT_ and Wzc_G569S_ (20). Others have reported that Wzc mutations increased visible colony mucoidy and total EPS in *rmp-*negative strains (26), while another study found that Wzc mutations increased sedimentation resistance, but not EPS, in both *rmp-*negative and *rmp*-positive strains, including a *K. variicola* isolate (55). Finally, another group isolated both WzcWT and Wzc variant-encoding *rmp-*positive strains from patients infected with CR-Kp; the strains containing Wzc variants had increased sedimentation resistance and thicker capsules based on EM imaging (56). Repeated isolation of Wzc variants under static culture conditions and from patient samples suggests that they may frequently arise within a population of bacteria and may expand when external conditions favor their unique properties, e.g., blocking phagocytosis (55). The observations made here, and by other groups (26, 55, 56), should serve as a warning that antibiotic-resistant, classical *K. pneumoniae* strains also harbor the potential to acquire the virulence-associated hypermucoid state by incorporating single-point mutations in Wzc.

How these Wzc variants change CPS chain length and cell-surface attachment, and how this alters mucoidy, remain to be defined. Wzc is often described as a molecular timer (38). In this process, de-phosphorylated Wzc monomers oligomerize and then phosphorylate the poly-tyrosine tail of the adjacent monomer (39). Once a critical capacity of Wzc phosphorylation is reached, the oligomer dissociates (23). This allows Wzb to de-phosphorylate Wzc and reinitiates the cycle, i.e., resets the timer (38, 57). The periodicity of Wzc oligomerization and dissociation controls CPS polymerization and secretion (23); our data suggest that it may also affect cell-surface anchoring. The Wzc variants identified here appear to not affect the total abundance of CPS by uronic acid quantification but rather alter the interval at which Wzc regulates the growing polysaccharide chain length.

While some identified Wzc variants reduce autophosphorylation status, not all do. Nonetheless, all identified Wzc variants eliminate the diverse type A polysaccharides and increase synthesis and release of uniform type B polysaccharides into the culture supernatant. Furthermore, Wzc_526insA_, Wzc_G569V_, and Wzc_P646S_, but not Wzc_Q395K_, boost production of ultra-high-molecular-weight type C CPS. Wzc_Q395K_ is notably the one Wzc variant that appears mucoid on solid medium yet has low sedimentation resistance (Fig. 4). Therefore, we propose that these Wzc variants all exert a similar impact on Wzc periodicity, some of which may be due to altered kinase/phosphatase dynamics but could also be due to altered active site dynamics, multimerization, or interactions with other binding partners (38). Furthermore, even though Wzc variants often increase release of CPS from the cell surface, we propose that mucoidy is driven by changes in the properties of cell-associated CPS (likely type C chains) since we observed increased mucoidy without increased CPS release in the pBAD18-overexpression system (Fig. 5).

In summary, our data support a model where *K. pneumoniae* regulates mucoidy in response to environmental cues and implicate Wzc-dependent changes in CPS chain length in controlling *K. pneumoniae* mucoidy. Our data have shown that Wzc acts downstream of RmpD to regulate mucoidy and does not necessarily increase CPS abundance. Conditions reported to increase mucoidy without increasing CPS abundance could be attributed to changes in CPS polymerization, which are not detected in bulk uronic acid quantification assays. Therefore, Wzc represents the lynchpin linking CPS biosynthesis and mucoidy.

## MATERIALS AND METHODS

### Bacterial strains and culture conditions

All primers, strains, and plasmids described in these studies are detailed in Tables S1 and S2. Bacteria were cultured in lysogeny broth (5 g/L yeast extract, 10 g/L tryptone, 0.5 g/L NaCl) at 200 rpm and 37°C unless otherwise noted. Solid medium was prepared by the addition of 20 g/L bacto-agar prior to autoclaving. When appropriate, antibiotics were added at the following concentrations: kanamycin (25 µg/mL), ampicillin (100 µg/mL), and chloramphenicol (20 µg/mL *E. coli* or 80 µg/mL *K*. *pneumoniae*). Human urine was collected anonymously from healthy women who were neither menstruating, pregnant, nor within 2 wk of antibiotic treatment. Urine was pooled from at least five independent donors and vacuum filtered and sterilized through 0.2 µm PES membrane. Sterilized urine was stored in 50 mL aliquots at −20°C, and working volumes were stored at 4°C. Solid urine medium was made by mixing warmed urine 1:1 with autoclaved bacto-agar (30 g/L) and then poured into Petri dishes.

### Uronic acid quantification

For experiments where bacteria were cultured in urine, 1 OD_600_ of bacteria was transferred to a microcentrifuge tube and pelleted at 21,000 *× g* for 15 min. The bacterial pellet was washed with 1 mL of PBS and centrifuged at 21,000 × *g* for 15 min. The washed cells were resuspended to 250 µL PBS, then 50 µL of 3–14 Zwittergent was added, and uronic acid quantification was performed as previously described (19, 58). For CPS quantification in all other media conditions, uronic acid quantification was performed as previously described (19, 58). Cell-free EPS was quantified by mixing 250 µL of the overnight culture with 50 µL of ultra-pure water instead of 3–14 Zwittergent. The culture was pelleted at 17,000 *× g* for 5 min, then 100 µL of the upper supernatant was transferred to 400 µL of cold ethanol to precipitate cell-free EPS, and uronic acid quantification was performed as previously described. CPS uronic acid content was deduced by subtracting the uronic acid levels in the whole culture from those in cell-free EPS.

### Sedimentation assay

Hypermucoviscosity was quantified using a sedimentation assay as previously described (19). In brief, 1 OD_600_ unit of bacteria cultured overnight was pelleted in a 2-mL microcentrifuge tube at 1,000 × *g* for 5 min. The OD_600_ of the supernatant was then quantified. If experimental conditions resulted in an overnight OD_600_ <1 (e.g., urine), 1 OD_600_ of bacteria was transferred to a 2-mL microcentrifuge tube and pelleted at 21,000 × *g* for 15 min. All but 100 µL of the sample supernatant was removed, then the bacterial pellet was resuspended to 1 mL with PBS, and sedimentation efficiency was assessed as described above.

### OD_600_ to CFU correlation

Colony-forming units (CFUs) were evaluated by correcting the overnight bacterial cultures to 2.0 and 0.2 OD _600_ units. Then, the bacterial cultures were serially diluted 10-fold in a microplate and spot-plated on LB agar plates at 20°C for 14 h. CFUs were counted and correlated with the OD_600_ units.

### RNA isolation and quantitative RT-PCR

qRT-PCR was performed as previously described with some modifications (59). In brief, bacteria cultured overnight in LB medium were sub-culture 1:100 into LB medium or 1:50 into sterile human urine. The bacteria were cultured with aeration at 37°C, 200 RPM for 2 h. Then, approximately 1 × 10^9^ CFU of bacteria were mixed at a 2:1 (vol:vol) ratio of RNAProtect (Qiagen) and incubated at room temperature for 5 min. Samples were then pelleted at 6,000 × *g* for 10 min, and the supernatant was drained.

RNA was purified with the RNeasy mini-prep kit (Qiagen) after lysozyme and proteinase K treatment for 40 min at room temperature. Samples were subjected to on-column DNase I treatment at room temperature for 20 min according to the manufacturer’s directions, eluting twice with 30 µL of RNase-free water. cDNA synthesis was performed with SuperScript III Reverse Transcriptase (Invitrogen) on an equal amount of RNA for each sample, roughly 3 µg total. The resulting cDNA was diluted 1:50 in water and utilized for real-time quantitative reverse transcription PCR (qRT-PCR) in a QuantStudio 3 PCR system (Applied Biosystem) with SYBRGreen PowerUp reagent (Invitrogen). Primers used to amplify internal fragments of CPS biosynthesis genes are listed in Table S1. The *gap2* transcript was used as an internal control. The relative fold change was calculated using the comparative threshold cycle (*C*_T_) method (60). PCR amplification efficiency controls were performed for each primer set, and dissociation curves were conducted to verify the amplification of a single product.

### Transposon screen in urine

The screen was performed similar to what was previously reported (19). Microplates containing the condensed, ordered library (total of 3,733 mutants) were thawed at room temperature and replicated into 100 µL of sterile-filtered pooled human urine in round bottom microplates. Plates were wrapped with plastic wrap to prevent evaporation and incubated statically at 37°C for 18–19 h. The sedimentation assay was adapted to a microplate format as follows. Plates were vortexed on low for 15 s, and then the total OD_600_ was recorded. Plates were centrifuged at 2,000 × *g* for 20 min, and then the upper 50 µL of supernatant was transferred to a new microplate to measure the OD_600_. Transposon mutants with a total OD_600_ more than two standard deviations from the plate mean were discarded. Transposon mutants with a supernatant OD_600_ more than two standard deviations from the plate mean were considered hits. The hits were struck onto urine agar, incubated at 37°C for 24 h, and then evaluated by string test. If a transposon mutant had a colony stretch more than 5 mm, it was identified as a candidate hit. To validate these candidates, three colonies of each transposon mutant were cultured in 3 mL of sterile urine overnight at 37°C with aeration, and then the OD_600_ of 100 µL of the total culture and culture supernatant was determined in a microplate before and after centrifugation at 7,000 × *g* for 10 min. Colonies with a supernatant OD_600_ greater than two standard deviations from the wild-type KPPR1 mean (0.0556) were considered hits. In some instances, individual isolates were considered positive by string test as colony heterogeneity was noted. Transposon clone IDs (Table 1) appended with a “g” were not colony purified until after the 3 mL sedimentation assay.

### Transposon site insertion validation

Bacterial lysates were used as genomic DNA template for PCR. Templates were prepared by inoculating 100 µL nuclease-free water with bacterial colonies, boiling the samples in the microwave for 1 min and then performing one freeze-thaw cycle. One microliter of cell lysate was used as template for a 25-µL PCR reaction using *Taq* DNA Polymerase with ThermoPol Buffer (NEB). For each mutant, one primer annealed to the transposon sequence and one primer annealed to the genomic region adjacent to the predicted transposon insertion site (Table S1). PCR amplification was confirmed on a 1% agarose gel.

### Whole-genome sequencing and analysis

Genomic DNA was purified from overnight cultures of each bacterial strain. One milliliter of bacterial culture was pelleted at 15,000 × *g* for 15 min at 4°C, and then samples were prepared according to the directions for the Wizard HMW DNA Extraction kit for Gram-negative Bacteria (Promega). The University of Michigan Microbiome Core quantified the genomic DNA samples, prepared the libraries using an Illumina DNA prep kit, and sequenced with a MiSeq Reagent Kit v3 (600-cycle). Sequence variants were detected using the variation analysis pipeline on PATRIC with the *K. pneumoniae* subsp. *pneumoniae* strain VK055 (RefSeq accession: NZ_CP009208.1; PATRIC Genome ID: 72407.38) as the target genome (35, 36). Parameters used were the BWA-mem Aligner and FreeBayes SNP Caller.

### *wzc* sequencing and analysis

Cell lysates were prepared as described above used as DNA template for PCR amplification using Phusion polymerase (NEB) and oligonucleotides that anneal 150 bp outside of *wzc*. For Sanger sequencing, PCR amplification was confirmed on a 1% agarose gel, DNA fragments column purified (Epoch), and Sanger sequenced with nested primers. For Oxford Nanopore sequencing, PCR amplification was confirmed on a 0.8% agarose gel, and the *wzc* PCR product was cloned into a TOPO vector using high-fidelity blunt-end TOPO Cloning (ThermoFisher). The TOPO vector with *wzc* insert was then transformed into chemically competent TOP10 cells and plated on LB agar containing kanamycin and X-gal (100 µg/mL). Plasmids were column purified (Epoch) and sequenced (Plasmidsaurus or Primordium Labs). Point mutations were identified using ClustalW (MegAlign. Version 15.3. DNASTAR. Madison, WI, USA) or multiple sequence alignment (Benchling. Version 1.5.1. San Francisco, CA, USA).

### Molecular cloning and transformation

Oligonucleotides and plasmids used in this study are listed in Tables S1 and S2. A 2 kb fragment encompassing 1 kb upstream and 1 kb downstream of the *wzc* stop codon was TOPO-TA cloned into pCR2.1 (Invitrogen). Each Wzc point mutation was introduced either by inverse PCR with overlapping mutagenic primers and self-ligation or by inverse PCR with 5′-phosphorylated, non-overlapping primers and T4 ligation. The pBAD18 backbone, *wzc* 5′ fragment, and mutated *wzc* 3′ fragment were PCR amplified, gel purified (Monarch, NEB), and assembled using NEBuilder HiFi DNA Assembly mix (NEB) for 1 h at 60°C (61). The ligated products were dialyzed on 0.025 µm MCE membranes (MilliporeSigma) against 10% glycerol prior to electroporation. A C-terminal hexa-histidine tag was added to each pBAD18-Wzc vector with 5′-phosphorylated, non-overlapping primers and T4 ligation. The integrity of each plasmid was confirmed by sequencing.

Electroporation of vectors into TOP10 *E. coli* or *K. pneumoniae* complex strains was performed as previously described (19). Insertional mutants were generated using λ Red recombineering adapted to *K. pneumoniae* as previously described (19, 62).

### Genetic characterization of clinical UTI isolates and Wzc alignment

Raw sequence reads of 616 (SRA accession: SRR6653995), 664 (SRA accession: SRR4115180), 714 (SRA accession: SRR4115178), and 1346 (SRA accession: SRR4115182) from NCBI database were assembled using PATRIC (https://www.bv-brc.org). Assembled sequences were uploaded to Pathogenwatch (https://pathogen.watch/) for species identification.

The CPS biosynthetic locus of each of the above SRA-deposited genomes was searched by BLAST using the highly conserved *galF* ORF from KPPR1. All ORFs in the nucleotide sequence containing *galF* and the 9,000 bp of DNA downstream were identified using the NCBI ORFfinder. The corresponding *wzc* ORF (approximately 2,100 bp) was confirmed by a BLAST search that confirmed the ORF to contain a tyrosine kinase. The ORFs were translated and aligned using Clustal Omega. The alignment was visualized using Jalview and Boxy SVG.

### Western blot

Whole cell lysates were prepared from overnight cultures of each bacterial strain. One milliliter of bacterial culture was pelleted at 21,000 × *g* for 15 min at 4°C, and samples were kept on ice until they were boiled. Bacterial pellets were resuspended in 250 µL of lysis buffer; lysis buffer was prepared fresh as follows: one cOmplete Mini, EDTA-free Protease Inhibitor Cocktail tablet (Roche) and one PhosSTOP tablet (Roche) dissolved in 7 mL BugBuster (MilliporeSigma). Each sample was sonicated in two, 5 s pulses with 50% amplitude and 5 s rest between each pulse. If samples were viscous, lysed cells were treated with nuclease for 1 h at 37°C. Nuclease was prepared fresh (4.9 mg ribonuclease A [Worthington] and 1 mg deoxyribonuclease [Worthington] dissolved in 960 µL of 1 M Tris pH 7.5, 20 µL of 1 M CaCl_2_, and 20 µL 1 M MgCl), and 12 µL was added to 250 µL of lysate. Protein concentration was quantified by BCA assay (Pierce). Samples were prepared at 1.4 µg/µL in 1× SDS loading buffer and boiled at 70°C for 3.5 min. In most instances, 14 µg of protein was resolved on 12% SDS-PAGE gels and transferred to nitrocellulose. Membranes were blocked with 5% BSA in TBS overnight, then washed with TBS-T, and probed with 1:2,500 mouse anti-phosphotyrosine PY20 (primary) or 1:5,000 mouse anti-His-Tag (primary) and then 1:5,000 goat anti-mouse IgG (H + L)-HRP (secondary) (SouthernBiotech). Blots were developed with ECL Western Blotting Substrate (Pierce) and imaged on a G:Box Imager (Syngene).

For any western blots involving samples cultured in urine, all samples were prepared at 1 µg/µL, and each lane was loaded with 10 μg of protein.

### Blot stripping and re-probing

Bound antibodies were stripped off the blots probed for phosphotyrosine or His-tag with fresh mild stripping buffer for 10 min, twice. One liter of stripping buffer was prepared by adding 15 g glycine, 1 g SDS, and 10 mL Tween-20 in water, and pH was adjusted to 2.2. Blots were then washed twice with PBS (10 min) and then twice with TBS-T (5 min each). Stripped blots were re-probed with 1:2,500 anti-GAPDH loading control (primary) antibody (Invitrogen) and 1:5,000 goat anti-mouse IgG IgG (H + L)-HRP (secondary) (SouthernBiotech). Blots were developed with ECL Western Blotting Substrate (Pierce) and imaged on a G:Box Imager (Syngene).

### Western blot quantification

Western blots were quantified using ImageJ version 1.53 K for Windows. In brief, an equal area of each band and GAPDH was measured, and then the lane background was subtracted from each value. The background-subtracted value of each band was normalized to its respective background-subtracted GAPDH value. The ratio of the normalized value of each mutant to wild type was plotted. Quantification shown in the graphs is the average of three independent replicates.

### Growth curves

Bacterial strains were cultured at 200 rpm overnight in triplicate in 3 mL of LB medium in a culture tube at 37°C. The bacteria were sub-cultured at a 1:1,000 dilution in 3 mL of LB medium and grown at 37°C, 200 rpm for 6 h. The cultures were normalized to an OD_600_ of 0.01 in LB medium, and then 100 µL was aliquoted into a microplate. One-hundred microliters of uninoculated LB medium were placed in each edge well to eliminate edge effects. A Cytation 5 (Biotek) was used to record the OD_600_ every 30 min for 12 h. Cultures were incubated at 37°C with continuous, orbital shaking [282 cpm (3 mm)].

### CPS chain length visualization

For cell-attached CPS, 1.5 OD_600_ of bacteria was transferred to a microcentrifuge tube and pelleted at 21,000 × *g* for 15 min. The bacterial pellet was washed with 1 mL of PBS and centrifuged at 21,000 × *g* for 15 min. The washed cells were resuspended to 250 µL PBS, and then 50 µL of 3–14 Zwittergent was added and incubated at 50°C for 20 min. Samples were then centrifuged at 17,000 × *g* for 5 min. One-hundred microliters of upper supernatant were precipitated in 400 µL cold ethanol. For cell-free EPS, 1.5 OD_600_ of bacteria was directly centrifuged at 17,000 × *g* for 5 min, and 100 µL of supernatant was precipitated with 400 µL of cold ethanol. Precipitated CPS or EPS was hydrated in 200 µL water. Twenty microliters of cell-attached CPS or EPS sample (3:1, sample:4× loading dye) and 15 µL of Precision Plus All Blue standard (Bio-Rad) were loaded on 4%–15% TGX stain-free precast gel (Bio-Rad). The gel was run for 4.5 h at 300 V on ice at 4°C. After electrophoresis, the gel was washed in 200 mL ultrapure water for 10 min, a total of five times. It was then stained in 0.1% alcian blue stain (0.1% wt/vol ThermoFisher Alcian Blue 8 GX in stain base solution) for 1 h with rocking. Stain base solution was prepared as 40% ethanol and 60% 20 mM sodium acetate, pH 4.75. After staining, gel was de-stained in stain base solution overnight with rocking. After background stain was sufficiently minimized, the gel was stained using Pierce Silver Stain Kit (ThermoFisher) and imaged on a Syngene G:box using the visible protein setting.

### Macrophage association assays

Cell association assays were performed similar to previous work, with the following modifications (19). Immortalized macrophage cells derived from wild-type mice (BEI Resources #NR-9456) were maintained in DMEM medium with L-glutamine, 4.5 g/L glucose and sodium pyruvate (Corning) supplemented with 10% heat-inactivated fetal calf serum (Corning), 100 U/mL penicillin, and 100 µg/mL streptomycin in an atmosphere of 5% CO_2_. Confluent cells (~3 × 10^5^ cells/well) in 24-well tissue culture dishes were washed with 1 mL of PBS, and then 1 mL of 3 × 10^6^ CFU/mL bacteria (MOI 10) in additive-free DMEM was added to each well. Samples were spun at 500 rpm (54 × *g*) for 5 min and then incubated at 37°C, 5% CO_2_ for 2 h, followed by an incubation at 4°C for 1 h. Samples were washed three times with PBS and then lysed with 1 mL of 0.2% Triton-X100 in PBS for 5 min. Input and cell-associated bacterial counts were determined by serial dilution and CFU enumeration on LB agar.

### Statistics

All replicates represent biological replicates and were replicated at least three times. All statistical analyses were computed in Prism 9.5.0 (GraphPad Software, La Jolla, CA, USA). For experiments comparing multiple groups, significance was calculated using two-way ANOVA with a Bonferroni post-test to compare specific groups. A one sample *t* test was applied when comparing two groups or a single group to a hypothetical value of 1.00. In all instances, results were considered significant if the *P* value was less than or equal to 0.0332.

## Data Availability

Whole-genome sequencing data are available on NCBI SRA under accession code PRJNA986550.

## SUPPLEMENTAL MATERIAL

Supplemental Figures msphere.00288-23-s0001.pdf Figures S1-S8.

Table S1 msphere.00288-23-s0002.xlsx Primers used in this study.

Table S2 msphere.00288-23-s0003.xlsx Strains used in this study.

ASM does not own the copyrights to Supplemental Material that may be linked to, or accessed through, an article. The authors have granted ASM a non-exclusive, world-wide license to publish the Supplemental Material files. Please contact the corresponding author directly for reuse.

## References

[1] Magill SS , Edwards JR , Bamberg W , Beldavs ZG , Dumyati G , Kainer MA , Lynfield R , Maloney M , McAllister-Hollod L , Nadle J , Ray SM , Thompson DL , Wilson LE , Fridkin SK . 2014. Multistate point-prevalence survey of health care–associated infections. N Engl J Med 370:1198–1208. doi:10.1056/NEJMoa1306801 24670166 PMC4648343

[2] Ikuta KS , Swetschinski LR , Robles Aguilar G , Sharara F , Mestrovic T , Gray AP , Davis Weaver N , Wool EE , Han C , Gershberg Hayoon A , Aali A , Abate SM , Abbasi-Kangevari M , Abbasi-Kangevari Z , Abd-Elsalam S , Abebe G , Abedi A , Abhari AP , Abidi H , Aboagye RG , Absalan A , Abubaker Ali H , Acuna JM , Adane TD , Addo IY , Adegboye OA , Adnan M , Adnani QES , Afzal MS , Afzal S , Aghdam ZB , Ahinkorah BO , Ahmad A , Ahmad AR , Ahmad R , Ahmad S , Ahmad S , Ahmadi S , Ahmed A , Ahmed H , Ahmed JQ , Ahmed Rashid T , Ajami M , Aji B , Akbarzadeh-Khiavi M , Akunna CJ , Al Hamad H , Alahdab F , Al-Aly Z , Aldeyab MA , Aleman AV , Alhalaiqa FAN , Alhassan RK , Ali BA , Ali L , Ali SS , Alimohamadi Y , Alipour V , Alizadeh A , Aljunid SM , Allel K , Almustanyir S , Ameyaw EK , Amit AML , Anandavelane N , Ancuceanu R , Andrei CL , Andrei T , Anggraini D , Ansar A , Anyasodor AE , Arabloo J , Aravkin AY , Areda D , Aripov T , Artamonov AA , Arulappan J , Aruleba RT , Asaduzzaman M , Ashraf T , Athari SS , Atlaw D , Attia S , Ausloos M , Awoke T , Ayala Quintanilla BP , Ayana TM , Azadnajafabad S , Azari Jafari A , B DB , Badar M , Badiye AD , Baghcheghi N , Bagherieh S , Baig AA , Banerjee I , Barac A , Bardhan M , Barone-Adesi F , Barqawi HJ , Barrow A , Baskaran P , Basu S , Batiha A-M , Bedi N , Belete MA , Belgaumi UI , Bender RG , Bhandari B , Bhandari D , Bhardwaj P , Bhaskar S , Bhattacharyya K , Bhattarai S , Bitaraf S , Buonsenso D , Butt ZA , Caetano dos Santos FL , Cai J , Calina D , Camargos P , Cámera LA , Cárdenas R , Cevik M , Chadwick J , Charan J , Chaurasia A , Ching PR , Choudhari SG , Chowdhury EK , Chowdhury FR , Chu D-T , Chukwu IS , Dadras O , Dagnaw FT , Dai X , Das S , Dastiridou A , Debela SA , Demisse FW , Demissie S , Dereje D , Derese M , Desai HD , Dessalegn FN , Dessalegni SAA , Desye B , Dhaduk K , Dhimal M , Dhingra S , Diao N , Diaz D , Djalalinia S , Dodangeh M , Dongarwar D , Dora BT , Dorostkar F , Dsouza HL , Dubljanin E , Dunachie SJ , Durojaiye OC , Edinur HA , Ejigu HB , Ekholuenetale M , Ekundayo TC , El-Abid H , Elhadi M , Elmonem MA , Emami A , Engelbert Bain L , Enyew DB , Erkhembayar R , Eshrati B , Etaee F , Fagbamigbe AF , Falahi S , Fallahzadeh A , Faraon EJA , Fatehizadeh A , Fekadu G , Fernandes JC , Ferrari A , Fetensa G , Filip I , Fischer F , Foroutan M , Gaal PA , Gadanya MA , Gaidhane AM , Ganesan B , Gebrehiwot M , Ghanbari R , Ghasemi Nour M , Ghashghaee A , Gholamrezanezhad A , Gholizadeh A , Golechha M , Goleij P , Golinelli D , Goodridge A , Gunawardane DA , Guo Y , Gupta RD , Gupta S , Gupta VB , Gupta VK , Guta A , Habibzadeh P , Haddadi Avval A , Halwani R , Hanif A , Hannan M , Harapan H , Hassan S , Hassankhani H , Hayat K , Heibati B , Heidari G , Heidari M , Heidari-Soureshjani R , Herteliu C , Heyi DZ , Hezam K , Hoogar P , Horita N , Hossain MM , Hosseinzadeh M , Hostiuc M , Hostiuc S , Hoveidamanesh S , Huang J , Hussain S , Hussein NR , Ibitoye SE , Ilesanmi OS , Ilic IM , Ilic MD , Imam MT , Immurana M , Inbaraj LR , Iradukunda A , Ismail NE , Iwu CCD , Iwu CJ , J LM , Jakovljevic M , Jamshidi E , Javaheri T , Javanmardi F , Javidnia J , Jayapal SK , Jayarajah U , Jebai R , Jha RP , Joo T , Joseph N , Joukar F , Jozwiak JJ , Kacimi SEO , Kadashetti V , Kalankesh LR , Kalhor R , Kamal VK , Kandel H , Kapoor N , Karkhah S , Kassa BG , Kassebaum NJ , Katoto PD , Keykhaei M , Khajuria H , Khan A , Khan IA , Khan M , Khan MN , Khan MA , Khatatbeh MM , Khater MM , Khayat Kashani HR , Khubchandani J , Kim H , Kim MS , Kimokoti RW , Kissoon N , Kochhar S , Kompani F , Kosen S , Koul PA , Koulmane Laxminarayana SL , Krapp Lopez F , Krishan K , Krishnamoorthy V , Kulkarni V , Kumar N , Kurmi OP , Kuttikkattu A , Kyu HH , Lal DK , Lám J , Landires I , Lasrado S , Lee S , Lenzi J , Lewycka S , Li S , Lim SS , Liu W , Lodha R , Loftus MJ , Lohiya A , Lorenzovici L , Lotfi M , Mahmoodpoor A , Mahmoud MA , Mahmoudi R , Majeed A , Majidpoor J , Makki A , Mamo GA , Manla Y , Martorell M , Matei CN , McManigal B , Mehrabi Nasab E , Mehrotra R , Melese A , Mendoza-Cano O , Menezes RG , Mentis A-F , Micha G , Michalek IM , Micheletti Gomide Nogueira de Sá AC , Milevska Kostova N , Mir SA , Mirghafourvand M , Mirmoeeni S , Mirrakhimov EM , Mirza-Aghazadeh-Attari M , Misganaw AS , Misganaw A , Misra S , Mohammadi E , Mohammadi M , Mohammadian-Hafshejani A , Mohammed S , Mohan S , Mohseni M , Mokdad AH , Momtazmanesh S , Monasta L , Moore CE , Moradi M , Moradi Sarabi M , Morrison SD , Motaghinejad M , Mousavi Isfahani H , Mousavi Khaneghah A , Mousavi-Aghdas SA , Mubarik S , Mulita F , Mulu GBB , Munro SB , Muthupandian S , Nair TS , Naqvi AA , Narang H , Natto ZS , Naveed M , Nayak BP , Naz S , Negoi I , Nejadghaderi SA , Neupane Kandel S , Ngwa CH , Niazi RK , Nogueira de Sá AT , Noroozi N , Nouraei H , Nowroozi A , Nuñez-Samudio V , Nutor JJ , Nzoputam CI , Nzoputam OJ , Oancea B , Obaidur RM , Ojha VA , Okekunle AP , Okonji OC , Olagunju AT , Olusanya BO , Omar Bali A , Omer E , Otstavnov N , Oumer B , P A M , Padubidri JR , Pakshir K , Palicz T , Pana A , Pardhan S , Paredes JL , Parekh U , Park E-C , Park S , Pathak A , Paudel R , Paudel U , Pawar S , Pazoki Toroudi H , Peng M , Pensato U , Pepito VCF , Pereira M , Peres MFP , Perico N , Petcu I-R , Piracha ZZ , Podder I , Pokhrel N , Poluru R , Postma MJ , Pourtaheri N , Prashant A , Qattea I , Rabiee M , Rabiee N , Radfar A , Raeghi S , Rafiei S , Raghav PR , Rahbarnia L , Rahimi-Movaghar V , Rahman M , Rahman MA , Rahmani AM , Rahmanian V , Ram P , Ranjha M , Rao SJ , Rashidi M-M , Rasul A , Ratan ZA , Rawaf S , Rawassizadeh R , Razeghinia MS , Redwan EMM , Regasa MT , Remuzzi G , Reta MA , Rezaei N , Rezapour A , Riad A , Ripon RK , Rudd KE , Saddik B , Sadeghian S , Saeed U , Safaei M , Safary A , Safi SZ , Sahebazzamani M , Sahebkar A , Sahoo H , Salahi S , Salahi S , Salari H , Salehi S , Samadi Kafil H , Samy AM , Sanadgol N , Sankararaman S , Sanmarchi F , Sathian B , Sawhney M , Saya GK , Senthilkumaran S , Seylani A , Shah PA , Shaikh MA , Shaker E , Shakhmardanov MZ , Sharew MM , Sharifi-Razavi A , Sharma P , Sheikhi RA , Sheikhy A , Shetty PH , Shigematsu M , Shin JI , Shirzad-Aski H , Shivakumar KM , Shobeiri P , Shorofi SA , Shrestha S , Sibhat MM , Sidemo NB , Sikder MK , Silva L , Singh JA , Singh P , Singh S , Siraj MS , Siwal SS , Skryabin VY , Skryabina AA , Socea B , Solomon DD , Song Y , Sreeramareddy CT , Suleman M , Suliankatchi Abdulkader R , Sultana S , Szócska M , Tabatabaeizadeh S-A , Tabish M , Taheri M , Taki E , Tan K-K , Tandukar S , Tat NY , Tat VY , Tefera BN , Tefera YM , Temesgen G , Temsah M-H , Tharwat S , Thiyagarajan A , Tleyjeh II , Troeger CE , Umapathi KK , Upadhyay E , Valadan Tahbaz S , Valdez PR , Van den Eynde J , van Doorn HR , Vaziri S , Verras G-I , Viswanathan H , Vo B , Waris A , Wassie GT , Wickramasinghe ND , Yaghoubi S , Yahya G , Yahyazadeh Jabbari SH , Yigit A , Yiğit V , Yon DK , Yonemoto N , Zahir M , Zaman BA , Zaman SB , Zangiabadian M , Zare I , Zastrozhin MS , Zhang Z-J , Zheng P , Zhong C , Zoladl M , Zumla A , Hay SI , Dolecek C , Sartorius B , Murray CJL , Naghavi M . 2022. Global mortality associated with 33 bacterial pathogens in 2019: a systematic analysis for the global burden of disease study 2019. Lancet 400:2221–2248. doi:10.1016/S0140-6736(22)02185-7 36423648 PMC9763654

[3] Foxman B . 2010. The epidemiology of urinary tract infection. Nat Rev Urol 7:653–660. doi:10.1038/nrurol.2010.190 21139641

[4] Fang C-T , Lai S-Y , Yi W-C , Hsueh P-R , Liu K-L , Chang S-C . 2007. Klebsiella pneumoniae genotype K1: an emerging pathogen that causes septic ocular or central nervous system complications from pyogenic liver abscess. Clin Infect Dis 45:284–293. doi:10.1086/519262 17599305

[5] Lee C-R , Lee JH , Park KS , Jeon JH , Kim YB , Cha C-J , Jeong BC , Lee SH . 2017. Antimicrobial resistance of hypervirulent Klebsiella pneumoniae: epidemiology, hypervirulence-associated determinants, and resistance mechanisms. Front Cell Infect Microbiol 7:483. doi:10.3389/fcimb.2017.00483 29209595 PMC5702448

[6] Codjoe FS , Donkor ES . 2017. Carbapenem resistance: a review. Med Sci 6:1. doi:10.3390/medsci6010001 PMC5872158 29267233

[7] Yigit H , Queenan AM , Anderson GJ , Domenech-Sanchez A , Biddle JW , Steward CD , Alberti S , Bush K , Tenover FC . 2001. Novel carbapenem-hydrolyzing beta-lactamase, KPC-1, from a carbapenem-resistant strain of Klebsiella pneumoniae. Antimicrob Agents Chemother 45:1151–1161. doi:10.1128/AAC.45.4.1151-1161.2001 11257029 PMC90438

[8] Munoz-Price LS , Poirel L , Bonomo RA , Schwaber MJ , Daikos GL , Cormican M , Cornaglia G , Garau J , Gniadkowski M , Hayden MK , Kumarasamy K , Livermore DM , Maya JJ , Nordmann P , Patel JB , Paterson DL , Pitout J , Villegas MV , Wang H , Woodford N , Quinn JP . 2013. Clinical epidemiology of the global expansion of Klebsiella pneumoniae carbapenemases. Lancet Infect Dis 13:785–796. doi:10.1016/S1473-3099(13)70190-7 23969216 PMC4673667

[9] Weiner LM , Webb AK , Limbago B , Dudeck MA , Patel J , Kallen AJ , Edwards JR , Sievert DM . 2016. Antimicrobial-resistant pathogens associated with healthcare-associated infections: summary of data reported to the national healthcare safety network at the centers for disease control and prevention, 2011-2014. Infect Control Hosp Epidemiol 37:1288–1301. doi:10.1017/ice.2016.174 27573805 PMC6857725

[10] Qureshi ZA , Syed A , Clarke LG , Doi Y , Shields RK . 2014. Epidemiology and clinical outcomes of patients with carbapenem-resistant Klebsiella pneumoniae bacteriuria. Antimicrob Agents Chemother 58:3100–3104. doi:10.1128/AAC.02445-13 24637691 PMC4068487

[11] Crosa JH , Walsh CT . 2002. Genetics and assembly line enzymology of siderophore biosynthesis in bacteria. Microbiol Mol Biol Rev 66:223–249. doi:10.1128/MMBR.66.2.223-249.2002 12040125 PMC120789

[12] Shon AS , Bajwa RPS , Russo TA . 2013. Hypervirulent (hypermucoviscous) Klebsiella pneumoniae. Virulence 4:107–118. doi:10.4161/viru.22718 23302790 PMC3654609

[13] Lam MMC , Wyres KL , Duchêne S , Wick RR , Judd LM , Gan Y-H , Hoh C-H , Archuleta S , Molton JS , Kalimuddin S , Koh TH , Passet V , Brisse S , Holt KE . 2018. Population genomics of hypervirulent Klebsiella pneumoniae clonal-group 23 reveals early emergence and rapid global dissemination. Nat Commun 9:2703. doi:10.1038/s41467-018-05114-7 30006589 PMC6045662

[14] Kim YJ , Kim SI , Kim YR , Wie SH , Lee HK , Kim S-Y , Park Y-J . 2017. Virulence factors and clinical patterns of hypermucoviscous Klebsiella pneumoniae isolated from urine. Infect Dis 49:178–184. doi:10.1080/23744235.2016.1244611 27829327

[15] Struve C , Roe CC , Stegger M , Stahlhut SG , Hansen DS , Engelthaler DM , Andersen PS , Driebe EM , Keim P , Krogfelt KA . 2015. Mapping the evolution of hypervirulent Klebsiella pneumoniae. mBio 6:e00630. doi:10.1128/mBio.00630-15 26199326 PMC4513082

[16] Palacios M , Broberg CA , Walker KA , Miller VL . 2017. A serendipitous mutation reveals the severe virulence defect of a Klebsiella pneumoniae fepB mutant. mSphere 2:e00341-17. doi:10.1128/mSphere.00341-17 28861522 PMC5566837

[17] Walker KA , Miner TA , Palacios M , Trzilova D , Frederick DR , Broberg CA , Sepúlveda VE , Quinn JD , Miller VL , Goldberg JB . 2019. A Klebsiella pneumoniae regulatory mutant has reduced capsule expression but retains hypermucoviscosity. mBio 10:e00089-19. doi:10.1128/mBio.00089-19 30914502 PMC6437046

[18] Walker KA , Treat LP , Sepúlveda VE , Miller VL , Heran Darwin K . 2020. The small protein RmpD drives hypermucoviscosity in Klebsiella pneumoniae. mBio 11:e01750-20. doi:10.1128/mBio.01750-20 32963003 PMC7512549

[19] Mike LA , Stark AJ , Forsyth VS , Vornhagen J , Smith SN , Bachman MA , Mobley HLT . 2021. A systematic analysis of hypermucoviscosity and capsule reveals distinct and overlapping genes that impact Klebsiella pneumoniae fitness. PLOS Pathog. 17:e1009376. doi:10.1371/journal.ppat.1009376 33720976 PMC7993769

[20] Ovchinnikova OG , Treat LP , Teelucksingh T , Clarke BR , Miner TA , Whitfield C , Walker KA , Miller VL . 2023. Hypermucoviscosity regulator RmpD interacts with Wzc and controls capsular polysaccharide chain length. mBio:e0080023. doi:10.1128/mbio.00800-23 37140436 PMC10294653

[21] Whitfield C . 2006. Biosynthesis and assembly of capsular polysaccharides in Escherichia coli. Annu Rev Biochem 75:39–68. doi:10.1146/annurev.biochem.75.103004.142545 16756484

[22] Wugeditsch T , Paiment A , Hocking J , Drummelsmith J , Forrester C , Whitfield C . 2001. Phosphorylation of Wzc, a tyrosine autokinase, is essential for assembly of group 1 capsular polysaccharides in Escherichia coli. J Biol Chem 276:2361–2371. doi:10.1074/jbc.M009092200 11053445

[23] Yang Y , Liu J , Clarke BR , Seidel L , Bolla JR , Ward PN , Zhang P , Robinson CV , Whitfield C , Naismith JH . 2021. The molecular basis of regulation of bacterial capsule assembly by Wzc. Nat Commun 12:4349. doi:10.1038/s41467-021-24652-1 34272394 PMC8285477

[24] Bushell SR , Mainprize IL , Wear MA , Lou H , Whitfield C , Naismith JH . 2013. Wzi is an outer membrane lectin that underpins group 1 capsule assembly in Escherichia coli. Structure 21:844–853. doi:10.1016/j.str.2013.03.010 23623732 PMC3791409

[25] Alvarez D , Merino S , Tomás JM , Benedí VJ , Albertí S . 2000. Capsular polysaccharide is a major complement resistance factor in lipopolysaccharide O side chain-deficient Klebsiella pneumoniae clinical isolates. Infect Immun 68:953–955. doi:10.1128/IAI.68.2.953-955.2000 10639470 PMC97229

[26] Ernst CM , Braxton JR , Rodriguez-Osorio CA , Zagieboylo AP , Li L , Pironti A , Manson AL , Nair AV , Benson M , Cummins K , Clatworthy AE , Earl AM , Cosimi LA , Hung DT . 2020. Adaptive evolution of virulence and persistence in carbapenem-resistant Klebsiella pneumoniae. Nat Med 26:705–711. doi:10.1038/s41591-020-0825-4 32284589 PMC9194776

[27] Martin RM , Cao J , Brisse S , Passet V , Wu W , Zhao L , Malani PN , Rao K , Bachman MA , Castanheira M . 2016. Molecular epidemiology of colonizing and infecting isolates of Klebsiella pneumoniae. mSphere 1:e00261-16. doi:10.1128/mSphere.00261-16 27777984 PMC5071533

[28] Broberg CA , Wu W , Cavalcoli JD , Miller VL , Bachman MA . 2014. Complete genome sequence of Klebsiella pneumoniae strain ATCC 43816 KPPR1, a rifampin-resistant mutant commonly used in animal, genetic, and molecular biology studies. Genome Announc 2:e00924-14. doi:10.1128/genomeA.00924-14 25291761 PMC4175196

[29] Wu KM , Li LH , Yan JJ , Tsao N , Liao TL , Tsai HC , Fung CP , Chen HJ , Liu YM , Wang JT , Fang CT , Chang SC , Shu HY , Liu TT , Chen YT , Shiau YR , Lauderdale TL , Su IJ , Kirby R , Tsai SF . 2009. Genome sequencing and comparative analysis of Klebsiella pneumoniae NTUH-K2044, a strain causing liver abscess and meningitis. J Bacteriol 191:4492–4501. doi:10.1128/JB.00315-09 19447910 PMC2704730

[30] Wyres KL , Wick RR , Gorrie C , Jenney A , Follador R , Thomson NR , Holt KE . 2016. Identification of Klebsiella capsule synthesis loci from whole genome data. Microb Genom 2:e000102. doi:10.1099/mgen.0.000102 28348840 PMC5359410

[31] Wick RR , Heinz E , Holt KE , Wyres KL . 2018. Kaptive web: user-friendly capsule and lipopolysaccharide serotype prediction for Klebsiella Genomes. J Clin Microbiol 56:e00197-18. doi:10.1128/JCM.00197-18 29618504 PMC5971559

[32] Lam MMC , Wick RR , Judd LM , Holt KE , Wyres KL . 2022. Kaptive 2.0: updated capsule and lipopolysaccharide locus typing for the Klebsiella pneumoniae species complex. Microb Genom 8:000800. doi:10.1099/mgen.0.000800 35311639 PMC9176290

[33] Holt KE , Wertheim H , Zadoks RN , Baker S , Whitehouse CA , Dance D , Jenney A , Connor TR , Hsu LY , Severin J , Brisse S , Cao H , Wilksch J , Gorrie C , Schultz MB , Edwards DJ , Nguyen KV , Nguyen TV , Dao TT , Mensink M , Minh VL , Nhu NTK , Schultsz C , Kuntaman K , Newton PN , Moore CE , Strugnell RA , Thomson NR . 2015. Genomic analysis of diversity, population structure, virulence, and antimicrobial resistance in Klebsiella pneumoniae, an urgent threat to public health. Proc Natl Acad Sci U S A 112:E3574–E3581. doi: 10.1073/pnas.1501049112 26100894 10.1073/pnas.1501049112 PMC4500264

[34] Lam MMC , Wick RR , Watts SC , Cerdeira LT , Wyres KL , Holt KE . 2021. A genomic surveillance framework and genotyping tool for Klebsiella pneumoniae and its related species complex. Nat Commun 12:4188. doi:10.1038/s41467-021-24448-3 34234121 PMC8263825

[35] Wattam AR , Davis JJ , Assaf R , Boisvert S , Brettin T , Bun C , Conrad N , Dietrich EM , Disz T , Gabbard JL , Gerdes S , Henry CS , Kenyon RW , Machi D , Mao C , Nordberg EK , Olsen GJ , Murphy-Olson DE , Olson R , Overbeek R , Parrello B , Pusch GD , Shukla M , Vonstein V , Warren A , Xia F , Yoo H , Stevens RL . 2017. Improvements to PATRIC, the all-bacterial bioinformatics database and analysis resource center. Nucleic Acids Res 45:D535–D542. doi:10.1093/nar/gkw1017 27899627 PMC5210524

[36] Davis JJ , Wattam AR , Aziz RK , Brettin T , Butler R , Butler RM , Chlenski P , Conrad N , Dickerman A , Dietrich EM , Gabbard JL , Gerdes S , Guard A , Kenyon RW , Machi D , Mao C , Murphy-Olson D , Nguyen M , Nordberg EK , Olsen GJ , Olson RD , Overbeek JC , Overbeek R , Parrello B , Pusch GD , Shukla M , Thomas C , VanOeffelen M , Vonstein V , Warren AS , Xia F , Xie D , Yoo H , Stevens R . 2020. The PATRIC bioinformatics resource center: expanding data and analysis capabilities. Nucleic Acids Res 48:D606–D612. doi:10.1093/nar/gkz943 31667520 PMC7145515

[37] Collins RF , Beis K , Dong C , Botting CH , McDonnell C , Ford RC , Clarke BR , Whitfield C , Naismith JH . 2007. The 3d structure of a periplasm-spanning platform required for assembly of group 1 capsular polysaccharides in Escherichia coli. Proc Natl Acad Sci U S A 104:2390–2395. doi:10.1073/pnas.0607763104 17283336 PMC1793899

[38] Hajredini F , Alphonse S , Ghose R . 2023. BY-kinases: protein tyrosine kinases like no other. J Biol Chem 299:102737. doi:10.1016/j.jbc.2022.102737 36423682 PMC9800525

[39] Bechet E , Gruszczyk J , Terreux R , Gueguen-Chaignon V , Vigouroux A , Obadia B , Cozzone AJ , Nessler S , Grangeasse C . 2010. Identification of structural and molecular determinants of the tyrosine-kinase Wzc and implications in capsular polysaccharide export. Mol Microbiol 77:1315–1325. doi:10.1111/j.1365-2958.2010.07291.x 20633230

[40] Hajredini F , Piserchio A , Ghose R . 2020. Long-range dynamic correlations regulate the catalytic activity of the bacterial tyrosine kinase Wzc. Sci Adv 6:eabd3718. doi:10.1126/sciadv.abd3718 33355134 PMC11206214

[41] Tan YH , Chen Y , Chu WHW , Sham L-T , Gan Y-H . 2020. Cell envelope defects of different capsule-null mutants in K1 hypervirulent Klebsiella pneumoniae can affect bacterial pathogenesis. Mol Microbiol 113:889–905. doi:10.1111/mmi.14447 31912541 PMC7317392

[42] Geisinger E , Isberg RR . 2015. Antibiotic modulation of capsular exopolysaccharide and virulence in Acinetobacter baumannii. PLOS Pathog 11:e1004691. doi:10.1371/journal.ppat.1004691 25679516 PMC4334535

[43] Kietzman CC , Gao G , Mann B , Myers L , Tuomanen EI . 2016. Dynamic capsule restructuring by the main pneumococcal autolysin lyta in response to the epithelium. Nat Commun 7:10859. doi:10.1038/ncomms10859 26924467 PMC4773454

[44] Hammerschmidt S , Wolff S , Hocke A , Rosseau S , Müller E , Rohde M . 2005. Illustration of pneumococcal polysaccharide capsule during adherence and invasion of epithelial cells. Infect Immun 73:4653–4667. doi:10.1128/IAI.73.8.4653-4667.2005 16040978 PMC1201225

[45] Murphy CN , Mortensen MS , Krogfelt KA , Clegg S . 2013. Role of Klebsiella pneumoniae type 1 and type 3 fimbriae in colonizing silicone tubes implanted into the bladders of mice as a model of catheter-associated urinary tract infections. Infect Immun 81:3009–3017. doi:10.1128/IAI.00348-13 23753626 PMC3719564

[46] Rosen DA , Pinkner JS , Jones JM , Walker JN , Clegg S , Hultgren SJ . 2008. Utilization of an intracellular bacterial community pathway in Klebsiella pneumoniae urinary tract infection and the effects of FimK on type 1 pilus expression. Infect Immun 76:3337–3345. doi:10.1128/IAI.00090-08 18411285 PMC2446714

[47] Rosen DA , Pinkner JS , Walker JN , Elam JS , Jones JM , Hultgren SJ . 2008. Molecular variations in Klebsiella pneumoniae and Escherichia coli FimH affect function and pathogenesis in the urinary tract. Infect Immun 76:3346–3356. doi:10.1128/IAI.00340-08 18474655 PMC2446687

[48] Struve C , Bojer M , Krogfelt KA . 2009. Identification of a conserved chromosomal region Encoding Klebsiella pneumoniae type 1 and type 3 fimbriae and assessment of the role of fimbriae in pathogenicity. Infect Immun 77:5016–5024. doi:10.1128/IAI.00585-09 19703972 PMC2772557

[49] Struve C , Bojer M , Krogfelt KA . 2008. Characterization of Klebsiella pneumoniae type 1 fimbriae by detection of phase variation during colonization and infection and impact on virulence. Infect Immun 76:4055–4065. doi:10.1128/IAI.00494-08 18559432 PMC2519443

[50] Simms AN , Mobley HLT . 2008. PapX, a P fimbrial operon-encoded inhibitor of motility in uropathogenic Escherichia coli. Infect Immun 76:4833–4841. doi:10.1128/IAI.00630-08 18710869 PMC2573324

[51] Hultgren SJ , Porter TN , Schaeffer AJ , Duncan JL . 1985. Role of type 1 pili and effects of phase variation on lower urinary tract infections produced by Escherichia coli. Infect Immun 50:370–377. doi:10.1128/iai.50.2.370-377.1985 2865209 PMC261959

[52] Gautam I , Huss CW , Storad ZA , Krebs M , Bassiouni O , Ramesh R , Wuescher LM , Worth RG . 2023. Activated platelets mediate monocyte killing of Klebsiella pneumoniae. Infect Immun 91:e0055622. doi:10.1128/iai.00556-22 36853027 PMC10016073

[53] Ventura CL , Cartee RT , Forsee WT , Yother J . 2006. Control of capsular polysaccharide chain length by UDP-sugar substrate concentrations in Streptococcus pneumoniae. Mol Microbiol 61:723–733. doi:10.1111/j.1365-2958.2006.05259.x 16780566

[54] Hudson AW , Barnes AJ , Bray AS , Ornelles DA , Zafar MA . 2022. Klebsiella pneumoniae L-fucose metabolism promotes gastrointestinal colonization and modulates its virulence determinants. Infect Immun 90:e0020622. doi:10.1128/iai.00206-22 36129299 PMC9584338

[55] Nucci A , Rocha EPC , Rendueles O . 2022. Adaptation to novel spatially-structured environments is driven by the capsule and alters virulence-associated traits. Nat Commun 13:4751. doi:10.1038/s41467-022-32504-9 35963864 PMC9376106

[56] He J , Shi Q , Chen Z , Zhang W , Lan P , Xu Q , Hu H , Chen Q , Fan J , Jiang Y , Loh B , Leptihn S , Zou Q , Zhang J , Yu Y , Hua X . 2023. Opposite evolution of pathogenicity driven by in vivo wzc and wcaJ mutations in ST11-KL64 carbapenem-resistant Klebsiella pneumoniae. Drug Resist Updat 66:100891. doi:10.1016/j.drup.2022.100891 36427451

[57] Alphonse S , Djemil I , Piserchio A , Ghose R . 2022. Structural basis for the recognition of the bacterial tyrosine kinase Wzc by its cognate tyrosine phosphatase Wzb. Proc Natl Acad Sci U S A 119:e2201800119. doi:10.1073/pnas.2201800119 35737836 PMC9245664

[58] Anderson MT , Mitchell LA , Zhao L , Mobley HLT . 2017. Capsule production and glucose metabolism dictate fitness during Serratia marcescens bacteremia. mBio 8:e00740-17. doi:10.1128/mBio.00740-17 28536292 PMC5442460

[59] Anderson MT , Mitchell LA , Mobley HLT . 2017. Cysteine biosynthesis controls Serratia marcescens phospholipase activity. J Bacteriol 199:e00159-17. doi:10.1128/JB.00159-17 28559296 PMC5527384

[60] Schmittgen TD , Livak KJ . 2008. Analyzing real-time PCR data by the comparative C_T_ method. Nat Protoc 3:1101–1108. doi:10.1038/nprot.2008.73 18546601

[61] Anderson MT , Himpsl SD , Mitchell LA , Kingsley LG , Snider EP , Mobley HLT . 2022. Identification of distinct capsule types associated with Serratia marcescens infection isolates. PLoS Pathog 18:e1010423. doi:10.1371/journal.ppat.1010423 35353877 PMC9000132

[62] Datsenko KA , Wanner BL . 2000. One-step inactivation of chromosomal genes in Escherichia coli K-12 using PCR products. Proc Natl Acad Sci U S A 97:6640–6645. doi:10.1073/pnas.120163297 10829079 PMC18686

