# Supplementary material for: Urine-mediated suppression of Klebsiella pneumoniae mucoidy is counteracted by spontaneous Wzc variants altering capsule chain length
Source: mSphere. 2023 Aug 23;8(5):e00288-23. doi: 10.1128/msphere.00288-23 (PMC10597399; doi:10.1128/msphere.00288-23)
Supplement: Supplemental Figures — Figures S1-S8. [file msphere.00288-23-s0001.pdf]

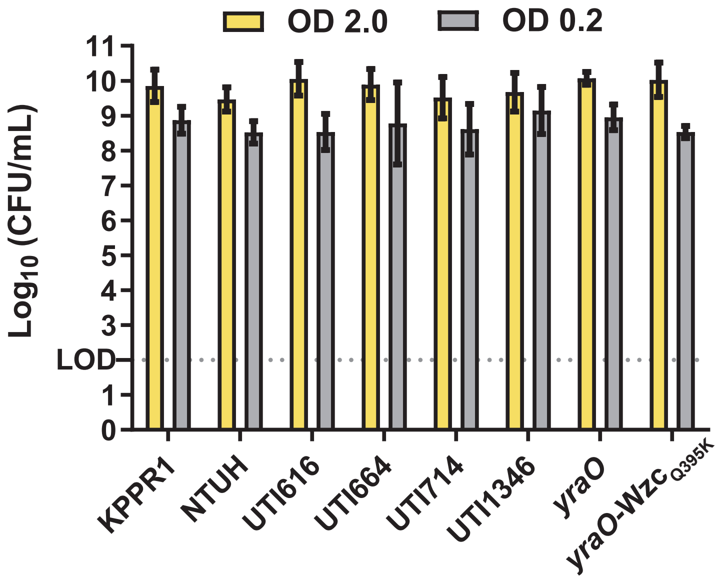

Figure S1. Correlation between CFU and OD_{600}. The relationship between CFU/mL and OD_{600} of key strains with dramatic differences in mucoidy or uronic acid content was assessed. Cells were cultured in LB overnight then normalized to OD 2.0 or 0.2 in PBS. Cells were serially diluted and enumerated on LB agar plates. Statistical significance was determined using one-way ANOVA with a Bonferroni post-test to compare each strain at the same OD_{600}. No significant differences were detected. Experiments were performed ≥3 independent times, in triplicate.

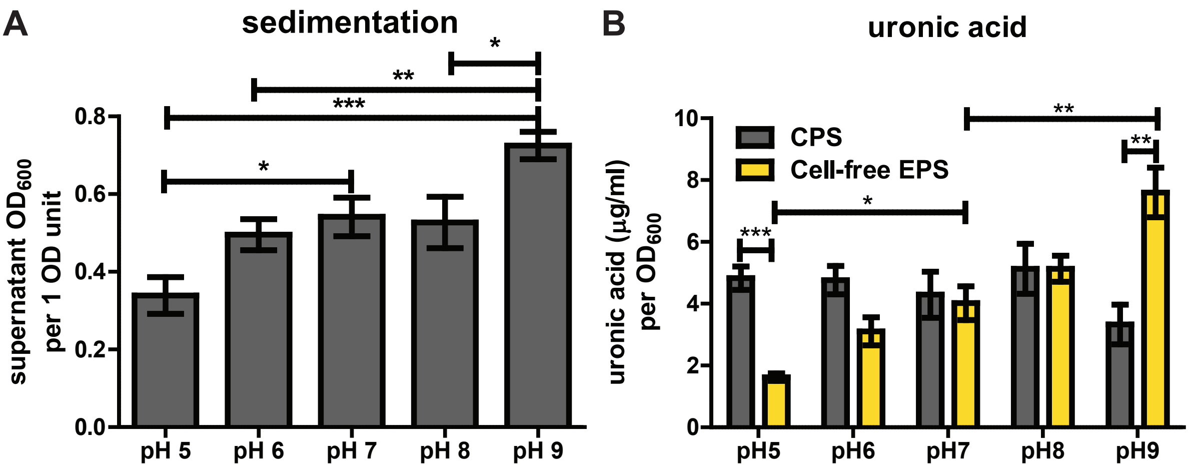

Figure S2. Medium alkalinity increases sedimentation resistance and cell-free extracellular polysaccharide (EPS) production. *K. pneumoniae* KPPR1 was cultured in pH-adjusted LB medium. (**A**) Mucoidy was determined by quantifying the supernatant OD600 after sedimenting 1 OD_{600} unit of culture at 1,000 x *g* for 5 min. (**B**) EPS was extracted from either total culture or spent medium and the uronic acid content was determined and normalized to the OD_{600} of the overnight culture. Statistical significance was determined using two-way ANOVA with a Bonferroni post-test to compare specific groups. * $p < 0.05$; ** $p < 0.01$; *** $p < 0.001$; # $p < 0.0001$. Experiments were performed ≥2 independent times, in triplicate.

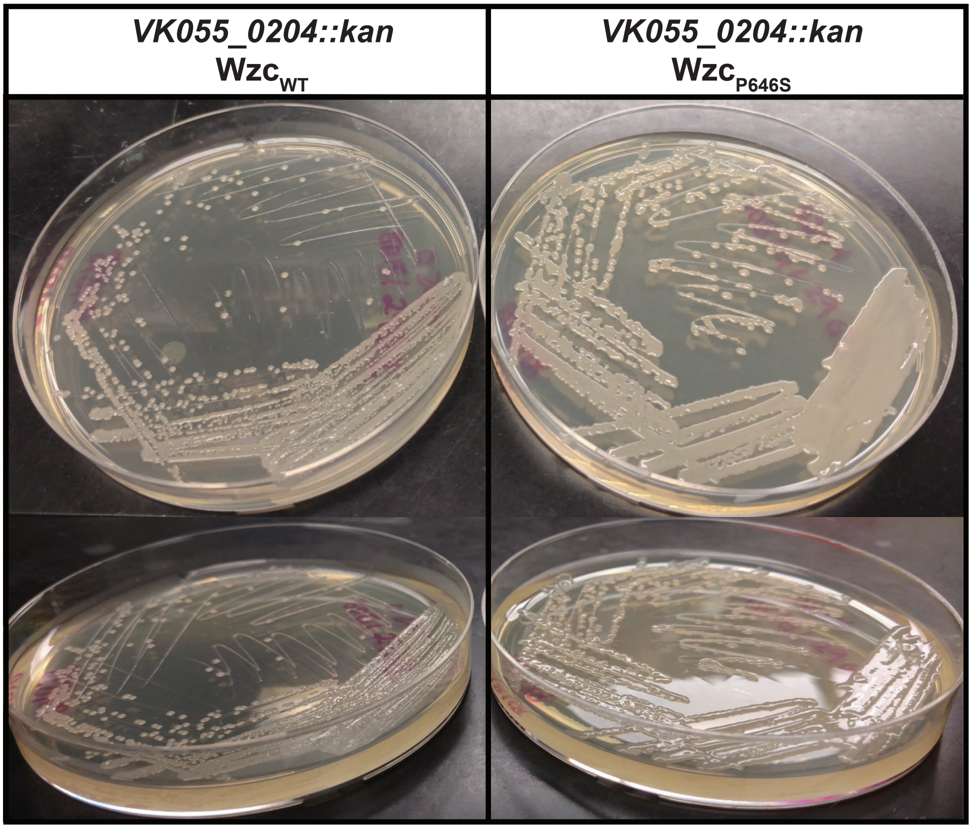

Figure S3. Phenotypic differences between spontaneous Wzc mutants on solid agar. Transposon strain *VK055_0204::kan* with Wzc wild type (WT) or a P646S variant were struck on LB agar and incubated at 30 ^{0}C overnight. Wzc variants have a distinct colony morphology that appears as larger, more translucent colonies, and colonies appear less distinct.

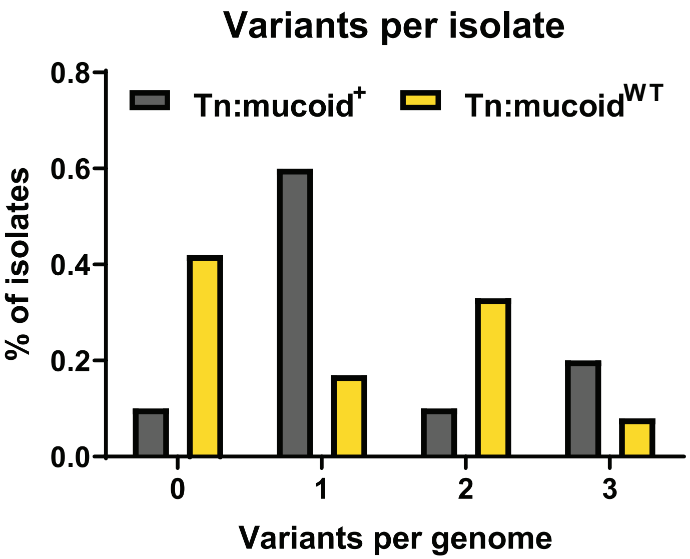

Figure S4. Transposon isolates with elevated mucoidy have increased frequency of single genetic variations. The genetic variation of 23 *K. pneumoniae* transposon isolates was determined using the Variation Analysis pipeline on PATRIC. The number of non-synonymous mutations per genome was plotted versus the frequency that number of mutations occurred in Tn:mucoid^{+} vs Tn:mucoid^{WT} isolates.

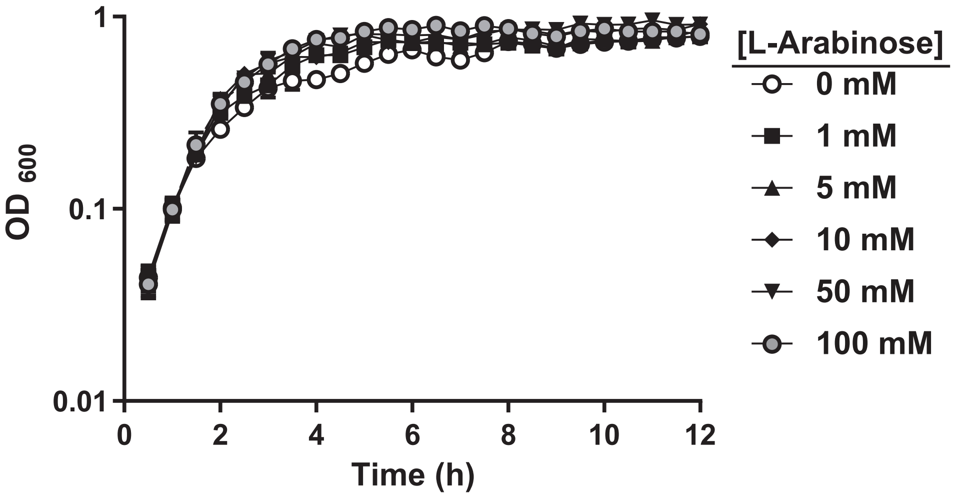

Figure S5. Impact of L-arabinose on KPPR1 growth in LB medium. Wild type *K. pneumoniae* strain KPPR1 was cultured in LB medium then back-diluted to OD_{600} 0.01 in LB medium with the indicated concentrations of L-arabinose. Cultures were incubated at 37 ^{0}C with continuous shaking. OD_{600} measurements were collected every 30 min. Growth assays were performed ≥3 independent times, in triplicate. Shown is the mean and error bars represent the standard error of the mean. No significant differences were detected using one-way ANOVA with a Dunnet post-test to compare each group to unmodified LB medium.

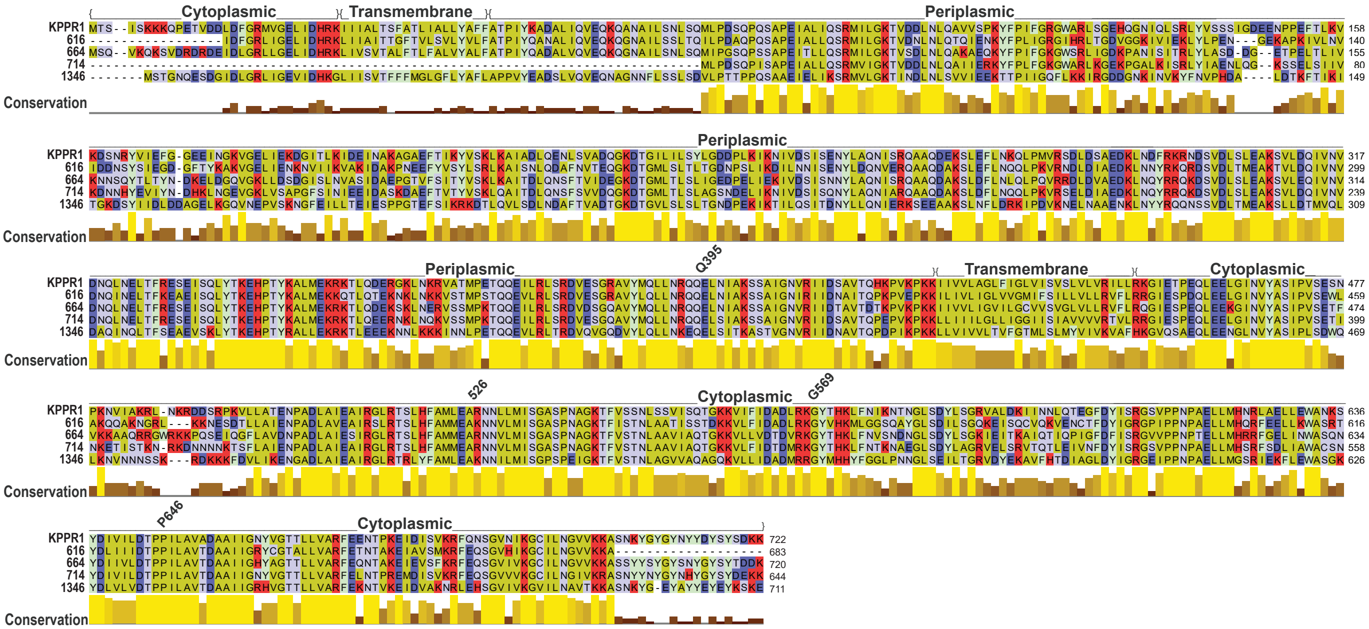

Figure S6. Wzc Protein Alignment. Predicted *wzc* open reading frames (ORFs) were extracted from corresponding unassembled genomes deposited in NCBI Sequence Read Archive. An alignment of the translated ORFs was generated with Clustal Omega and visualized with the Jalview application and the online Boxy SVG applet. Key mutation sites are indicated. The color of each amino acid represents basic (red), acidic (dark blue), polar uncharged (light blue), aromatic (green), and aliphatic (yellow) residues. Conservation between strains is indicated by the histograms below the alignments.

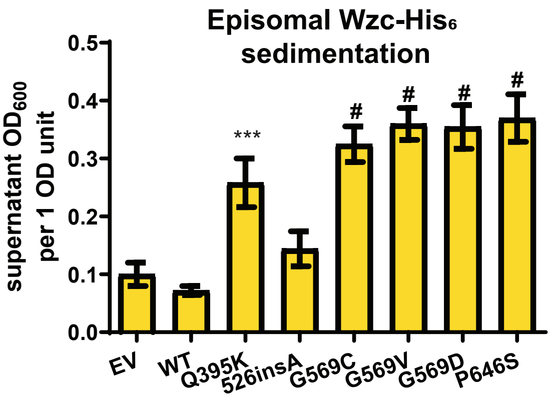

Figure S7. Sedimentation resistance of episomal Wzc-His$_6$ variants. KPPR1 transformed with His$_6$-tagged Wzc (WT, Q395K, 526insA, G569C, G569V, G569D, P646S) or empty vector pBAD18 (EV) was cultured in LB medium containing kanamycin with 50 mM L-arabinose. Mucoidy was determined by quantifying the supernatant OD_{600} after sedimenting 1 OD_{600} unit of culture at 1,000 x *g* for 5 min. Data presented are the mean and error bars represent the standard error of the mean. Statistical significance was determined using two-way ANOVA with a Bonferroni post-test to compare specific groups. # $p < 0.0001$. Experiments were performed ≥3 independent times, in triplicate.

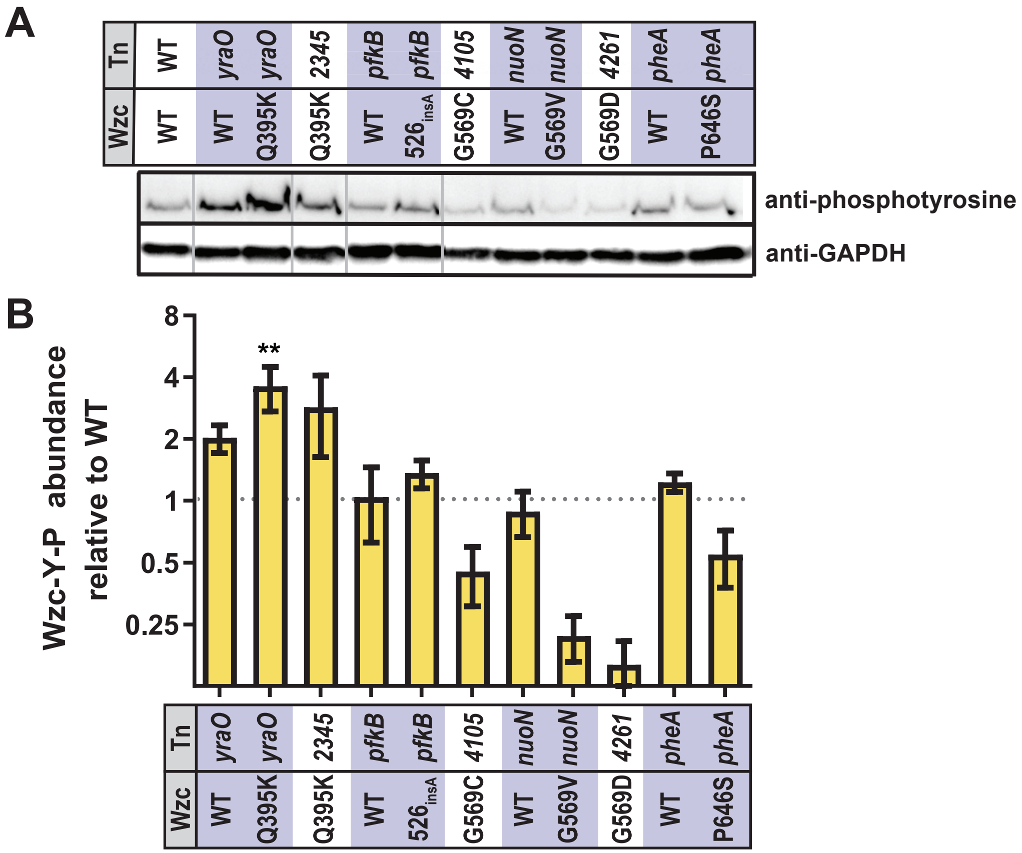

Figure S8. Wzc tyrosine phosphorylation status of transposon mutants with and without Wzc variants. The indicated strains were cultured in LB medium. (**A**) Whole cell lysates were resolved by SDS-PAGE, transferred to nitrocellulose, then probed with anti-phosphotyrosine antibody (PY20). Membranes were stripped and re-probed with anti-GAPDH (GA1R). Vertical gray lines indicate where gel lanes are rearranged to improve data presentation. (**B**) The Wzc-Y-P bands in A were quantified in ImageJ and normalized to GAPDH band intensity. Normalized Wzc-Y-P in each mutant was divided by the normalized Wzc-Y-Pi of wild type (WT) KPPR1 within each blot. In all instances lysates were prepared and analyzed ≥ 3 independent times. One representative image is shown. A one-way ANOVA with a Bonferroni post-test was used to determine if each mean was significantly different from 1.0 (WT), where ** $p < 0.0021$.
